# De Novo Design and Experimental Characterization of Ultrashort Self-Associating Peptides

**DOI:** 10.1371/journal.pcbi.1003718

**Published:** 2014-07-10

**Authors:** James Smadbeck, Kiat Hwa Chan, George A. Khoury, Bo Xue, Robert C. Robinson, Charlotte A. E. Hauser, Christodoulos A. Floudas

**Affiliations:** 1 Department of Chemical and Biological Engineering, Princeton University, Princeton, New Jersey, United States of America; 2 Institute of Bioengineering and Nanotechnology, Singapore, Singapore; 3 Institute of Molecular and Cell Biology, A*STAR (Agency of Science, Technology and Research), Biopolis, Singapore, Singapore; Stanford University, United States of America

## Abstract

Self-association is a common phenomenon in biology and one that can have positive and negative impacts, from the construction of the architectural cytoskeleton of cells to the formation of fibrils in amyloid diseases. Understanding the nature and mechanisms of self-association is important for modulating these systems and in creating biologically-inspired materials. Here, we present a two-stage *de novo* peptide design framework that can generate novel self-associating peptide systems. The first stage uses a simulated multimeric template structure as input into the optimization-based Sequence Selection to generate low potential energy sequences. The second stage is a computational validation procedure that calculates Fold Specificity and/or Approximate Association Affinity (K^*^_association_) based on metrics that we have devised for multimeric systems. This framework was applied to the design of self-associating tripeptides using the known self-associating tripeptide, Ac-IVD, as a structural template. Six computationally predicted tripeptides (Ac-LVE, Ac-YYD, Ac-LLE, Ac-YLD, Ac-MYD, Ac-VIE) were chosen for experimental validation in order to illustrate the self-association outcomes predicted by the three metrics. Self-association and electron microscopy studies revealed that Ac-LLE formed bead-like microstructures, Ac-LVE and Ac-YYD formed fibrillar aggregates, Ac-VIE and Ac-MYD formed hydrogels, and Ac-YLD crystallized under ambient conditions. An X-ray crystallographic study was carried out on a single crystal of Ac-YLD, which revealed that each molecule adopts a β-strand conformation that stack together to form parallel β-sheets. As an additional validation of the approach, the hydrogel-forming sequences of Ac-MYD and Ac-VIE were shuffled. The shuffled sequences were computationally predicted to have lower K^*^_association_ values and were experimentally verified to not form hydrogels. This illustrates the robustness of the framework in predicting self-associating tripeptides. We expect that this enhanced multimeric *de novo* peptide design framework will find future application in creating novel self-associating peptides based on unnatural amino acids, and inhibitor peptides of detrimental self-aggregating biological proteins.

## Author Summary

The self-association of peptides and proteins plays an important role in many serious diseases, such as Alzheimer's disease. A complete understanding of how peptides and proteins self-associate is important in creating therapeutics for such diseases. Additionally, self-associating peptides can be used as templates for bioinspired nanomaterials. With these goals in mind, we have proposed a de novo peptide design methodology capable of producing peptides that self-associate. We have experimentally tested the framework through the design of several self-associating tripeptides. Using the framework we designed six self-associating peptides, including two peptides, Ac-MYD and Ac-VIE, which readily formed hydrogels and one peptide, Ac-YLD, which readily formed a crystal. An X-ray crystallographic study was performed on Ac-YLD to determine its crystal structure. The top-ranked designed sequences were shuffled and computationally and experimentally characterized in order to validate that the approach can differentiate the self-associating of tripeptides, which are derived from the same amino acids. Through the analysis of the experimental results we determine which metrics are most important in the self-association of peptides. Additionally, the crystallographic structure of the tripeptide Ac-YLD provides a structural template for future self-association design experiments.

## Introduction

In nature, proteins and peptides self-assemble and associate to produce a variety of diverse structures such as cellular nanomachines and multimeric structures, including cellular pumps, cytoskeletal filaments, and fibrils [1]. These complex biological structures can serve as templates for the design of novel bioinspired nanomaterials, as well as for the exploration of the underlying mechanisms of self-assembly [2], [3]. The self-assembly of proteins is associated with the formation of amyloid fibrils that is implicated in the onset of Alzheimer's disease and other degenerative diseases [3]–[6]. While the causes of the onset of the formation of the disruptive fibrillar macrostructure has been well studied, the exact mechanism of self-assembly is not fully understood [6], [7]. It is known that even in large self-assembling peptides, the association can be driven by only a few key interacting residues [8]–[12]. For this reason, the de novo design and discovery of small peptides that self-assemble will have major implications for the understanding of the determinants of self-assembly, as well as for providing insights that can be used to disrupt such associations.

In addition to the medical relevance of self-assembling peptides and proteins, self-assembly in nature provides interesting and potentially fruitful avenues for biomaterial production, a field that has been amply covered in a variety of reviews [1], [13]–[25]. Small, self-assembling peptide structures are of particular interest as they are relatively inexpensive to produce by standard chemical synthesis [26] and provide tunability of properties through substitution of individual amino acids [27]–[29]. This allows for a “bottom-up” approach to creating novel self-assembled biomaterials [19], [20]. Several notable small associating peptides have been discovered by derivation of natural systems (e.g., Alzheimer's β-amyloid protein) and through rational design [13], [14], [25].

The design of self-assembling peptides for biomedical and biomaterial purposes has most commonly been performed through rational design and large-scale screening. The discovery of a self-assembling dipeptide [30]–[32] has demonstrated the applicability of methods to such a problem. However, the size of the peptide is limiting in this design process, since the immense sequence space (20^N^ possible designed sequences, where N is the number of design positions) that must be searched may, in many cases, overstretch the combinatorial capabilities of such experimental methods. Due to the considerable cost and time involved in synthesizing and testing a large number of candidate peptides, it is highly desirable to screen computationally for self-assembly properties prior to experimental testing of peptides. For this reason, the application of computational methods to the design of self-assembling peptides is highly desirable.

Computational protein design methods have become increasingly prevalent in the field of protein engineering. These design methods include those that employ probabilistic algorithms like Monte Carlo (MC) methods [33]–[36] and genetic algorithms [37], as well as deterministic algorithms like dead end elimination (DEE) [38]–[42], self-consistent mean field (SCMF) methods [43]–[48], or quadratic assignment-like global optimization for sequence selection followed by fold specificity and approximate binding affinity [49]–[55]. Such computational methods allow for the consideration of large numbers of amino acid-amino acid interactions simultaneously. Computational design has been used to design inhibitors against H1N1 influenza hemagglutinin [56], to switch cofactor specificity of an enzyme [57], for generalized antibody design for recognition of a target epitope [58], for the design of entry inhibitors of HIV-1 gp41 [59], for the design of C3a receptor agonists for medicinal use [54], and for the design of inhibitors of the histone methyltransferase EZH2 [55]. See Fung et al. [51], Pantazes et al. [60], Samish et al. [61], and Khoury et al. [62] for reviews of the recent advances and successes in the area.

As computational methods for single peptides and protein-peptide complexes have improved, the general interest in the design of multimeric protein assemblies for therapeutic and biomaterial applications [63]–[65] has also increased. Recently, there have been a number of successful computational designs carried out to create unique multimeric protein structures [66]–[69], Here we present a *de novo* protein/peptide design framework applicable to multimeric systems and its application to the design of self-associating tripeptides. This framework utilizes a computationally-generated multimeric assembly [51], [70] of the self-assembling tripeptide Ac-IVD [11] as the template for an optimization-based Sequence Selection method [49], [50], [71], [72]. Selected sequences are then computationally screened via a Fold Specificity calculation [70] and/or calculation of Association Affinity via molecular dynamics (MD) simulations. The Association Affinity metric is based on statistical mechanics [59], [73] and is used to select a small set of high confidence peptide sequences from the candidate set. To experimentally validate the framework, six *in silico* designed sequences were selected for experimental assessment based on the metrics described. We found that two of these tripeptides (Ac-VIE, Ac-MYD) formed hydrogels on time scales and at concentrations comparable to the template peptide Ac-IVD. Shuffled control sequences of these designed hydrogelating peptides were further experimentally and computationally assessed to validate the approach. Remarkably, Ac-YLD was capable of rapidly associating into large crystals under ambient conditions, which led to the elucidation of its crystal structure. The structural data obtained from the crystal are invaluable in refining the framework for improved accuracy in the design of self-associating systems.

## Results

### Computational Results

The outcomes of the optimization and simulation (Stages One and Two) are tabulated in Tables 1–3 (full results provided in Table S1). The Sequence Selection table shows that there is a high frequency of double aromatic residues (Trp, Tyr) present in the top ten sequences exhibiting the lowest potential energies (Pot.E), whereas there is a high frequency of Met and Ile being present in the last ten tripeptides with the highest potential energies (Table S2). Stage One calculates the pairwise interaction energies between residues. A fully extended polypeptide chain would result in side-chains of adjacent residues being on opposite planes of the polypeptide backbone. The fact that double aromatic residue sequences have been calculated to possess the lowest potential energies suggests that the backbones of these tripeptides are twisted to promote pairwise interactions between residues. Aromatic residues are known to associate via π-π/CH-π stacking, a prominent example being diphenylalanine [30]–[32], so the high ranking of double aromatic residue sequences (lowest potential energies) enhances confidence in Stage One results. The high frequency of linear aliphatic residues (Met, Ile) in the sequences of highest potential energies reflects that van der Waals interactions between adjacent aliphatic side-chains of Met/Ile are weak compared to the aromatic residues.

**Table 1:** Summary of results from Stage One: Sequence Selection.

Seq. Sel. Rank	P1	P2	P3	Potential Energy
1	TRP	TRP	ASP	−0.1255
2	TRP	TRP	GLU	−0.1193
3	TRP	TYR	GLU	−0.0912
4	TRP	TYR	ASP	−0.0851
5	TYR	TRP	GLU	−0.0801
6	TYR	TRP	ASP	−0.0788
7	TRP	LEU	GLU	−0.0755
8	TRP	LEU	ASP	−0.0687
9	LEU	TRP	ASP	−0.0664
10	LEU	TRP	GLU	−0.0663

The top ten candidates, ranked by potential energy (Pot.E), are given in the table.

**Table 2:** Summary of results for Run 1.

Seq. Sel. Rank	Pot.E	FSpec Rank	FSpec	K^*^_association_ Rank	K^*^_association_	Sequence
54*	−0.0324	4	6.07	1	1.66E-03	LVE
49*	−0.0340	7	5.18	2	4.97E-50	YLD
55	−0.0323	8	5.17	3	1.12E-54	VLE
48	−0.0342	19	2.66	4	1.34E-61	YIE
93*	−0.0173	18	2.69	5	5.39E-64	VIE
12*	−0.0618	13	3.54	6	4.31E-64	LLE
46*	−0.0361	10	3.89	7	2.65E-70	YYD
33	−0.0367	20	2.39	8	2.67E-72	LYD
27	−0.0483	1	8.48	9	1.13E-79	YLE
120	−0.0051	14	3.02	10	4.23E-90	VME

The top twenty sequences by Fold Specificity were re-ranked by Approximate Association Affinity. The top ten candidates after re-ranking are shown in the table. Five of the top sequences (*) were chosen from this re-ranked set for experimental validation.

**Table 3:** Summary of results for Run 2.

Seq. Sel. Rank	Pot.E	FSpec Rank	FSpec	K^*^_association_ Rank	K^*^_association_	Sequence
54*	−0.0324	4	6.07	1	1.66E-03	LVE
101**	−0.0151	35	1.37	2	3.05E-15	MYD
103	−0.0150	66	0.71	3	1.33E-24	AAD
47	−0.0349	62	0.84	4	3.97E-30	IYE
61	−0.0304	86	0.43	5	2.23E-30	IYD
100*	−0.0153	51	1.00	6	4.87E-32	IVD
20	−0.0517	70	0.65	7	7.80E-33	WVD
73	−0.0272	85	0.44	8	1.04E-33	LAD
33	−0.0467	111	0.18	9	8.25E-36	AWD
1	−0.1255	126	0.06	10	2.15E-36	WWD

The top ten sequences ranked by Approximate Association Affinity (K^*^_association_) from the 109-peptide set. The top sequence not chosen in Run 1 was chosen from this re-ranked set for experimental validation. The newly chosen peptide sequence is highlighted with (**). Previously selected peptides are highlighted with (*).

In order to improve confidence in the sequences to be selected for experimental validation, the full set of sequences was screened by Fold Specificity (FSpec) and ranked again. In turn, the top twenty sequences ranked by Fold Specificity were also assessed by the Approximate Association Affinity metric, K^*^_association_. This double-ranked set of peptide sequences is shown as “Run 1” in Table 2.

In order to separately assess the capabilities of the newly developed metric, 109 of the 128 tripeptide candidates were also directly assessed by K^*^_association_. 19 peptides were excluded since their Sequence Selection and/or Fold Specificity rankings were among the lowest ranking and thus did not warrant re-evaluation. The set of top ten sequences using this metric is given as “Run 2” in Table 3. Unlike for Sequence Selection in which sequences with double aromatic residues dominate the top of the rank, no outstanding trends were observed with regard to the residues of the top-ranked sequences for either Fold Specificity or Approximate Association Affinity, despite the expectation that sequences with aromatic residues might exhibit higher association affinity. This illustrates the ability of the Approximate Association Affinity metric to discern tripeptides that are strong candidates for association, but would have otherwise been difficult to identify through rational design.

The top-ranked tripeptide in both Runs 1 and 2, Ac-LVE, was selected for validation. Compared to Ac-LVE, Ac-YLD has similar Pot.E and FSpec, but different K^*^_association_, so it was also selected. Similarly, Ac-LLE and Ac-YYD were selected because they have similar FSpec and K^*^_association_, but different Pot.E. The Ac-LVE/Ac-YLD and Ac-LLE/Ac-YYD pairings might allow the respective effects of K^*^_association_ and Pot.E on self-association outcomes to be discerned. Lastly, Ac-MYD and Ac-VIE were selected as they have similar Pot.E to Ac-IVD. This allows the effects of FSpec and K^*^_association_ on hydrogelation to be assessed. Thus, the tripeptides chosen for experimental validation were Ac-IVD, Ac-LVE, Ac-YYD, Ac-LLE, Ac-YLD, Ac-MYD, and Ac-VIE (Table 4).

**Table 4:** Summary of peptides chosen for experimental validation.

Pot.ERank	Pot.E	FSpec Rank	FSpec	K^*^_association_	Seq.	Run	Peptide property	Exp. Observation	Storage modulus (G′, kPa)	Loss modulus (G″, kPa)
54*	−0.0324	4	6.07	1.66E-03	LVE	1,2	aliphatic	hydrogel	N.D.	N.D.
101**	−0.0151	35	1.37	3.05E-15	MYD	2	aromatic	hydrogel	20	9
100*	**−0.0153**	**51**	**1**	**4.87E-32**	**IVD**	**2**	**aliphatic**	**hydrogel** [87]	**3** [87]	**N.A.**
49*	−0.0340	7	5.18	4.97E-50	YLD	1	aromatic	crystal	N.D.	N.D.
93*	−0.0173	18	2.69	5.39E-64	VIE	1	aliphatic	hydrogel	8	1
12*	−0.0618	13	3.54	4.31E-64	LLE	1	aliphatic	bead-like structure	N.D.	N.D.
46*	−0.0361	10	3.89	2.65E-70	YYD	1	aromatic	fibrillar structure	N.D.	N.D.

Sequences are ranked in order of K^*^_association_ value. Template sequence known to hydrogel, Ac-IVD, is highlighted in bold.

It should be noted that the Fold Specificity and Approximate Association Affinity are used strictly as metrics for selecting which peptides should be experimentally tested. We are not attempting to compare the calculated values to exact, experimental Fold Specificity or Association Affinity values. Rather, we aim to produce metrics capable of ranking a set of peptides to increase the probability that the top ranked peptides are positive hits, in this case, self-associating peptides. For this reason, it is of little concern whether the properties of the produced peptides match exactly the ranking shown in the tables.

The inter-peptide interactions that are observed in the simulations of favorably self-associating sequences are predicted to have a higher tendency to self-associate (form hydrogels or crystals) and this forms the hypothesis being tested in this work. In the computational calculations, there is currently no metric that can distinguish whether the peptides could potentially form crystals or hydrogels.

### Experimental Validation of Designed Tripeptides: Self-Association and Rheological Studies

The six high-ranking tripeptides that were chosen to be evaluated based on their predicted abilities to self-associate can be divided into two classes: (1) the aliphatic class of Ac-LVE, Ac-VIE, Ac-LLE and (2) the aromatic class of Ac-MYD, Ac-YLD, and Ac-YYD. The ability of the tripeptides to associate was assessed across a concentration range from 5 mg/mL to the upper limit of 40 mg/mL, in steps of 5 mg/mL. Such a concentration series enables one to compare the association properties of the evaluated tripeptides at 20 mg/mL (concentration at which the simulations were run), as well as bracket the concentration in which there is a change in the association state of the tripeptide.

**Figure 1:**
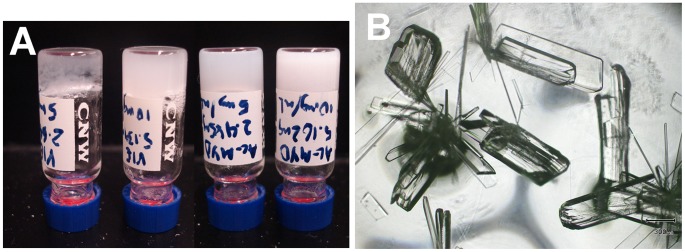
Experimental characterization of successful self-associating peptide design. (A) (From left to right) Hydrogels formed from: Ac-VIE, 5 mg/mL; Ac-VIE, 10 mg/mL; Ac-MYD, 5 mg/mL; Ac-MYD, 10 mg/mL (B) Crystals of Ac-YLD viewed under a light microscope.

Of the three aliphatic tripeptides, the top-ranked sequence, Ac-LVE, was able to form a gelatinous precipitate between 5 and 10 mg/mL. This precipitation persisted up to 30 mg/mL, with hydrogelation of Ac-LVE observed at 35 and 40 mg/mL. The second-ranked sequence, Ac-VIE was able to form a hydrogel at 5 mg/mL over 48 h; at 10 mg/mL, hydrogelation proceeded within 10 min (Figure 1A). The third-ranked sequence, Ac-LLE, formed a clear solution up to 40 mg/mL, even after standing for two weeks. This indicates that either there is no self-association, or that any association formed by Ac-LLE is still soluble in water.

Of the three aromatic tripeptides, the top-ranked sequence, Ac-MYD, was able to form a hydrogel at 5 mg/mL over 24 h; at 10 mg/mL, hydrogelation proceeded within 1 min (Figure 1A). The second-ranked sequence, Ac-YLD, spontaneously crystallized in water, even at the lowest concentration of 5 mg/mL, to furnish large crystals of diffraction quality under ambient conditions (Figure 1B). This indicates that the self-association of Ac-YLD proceeded in an orderly manner to produce the well-defined packing of a crystal. The third-ranked sequence, Ac-YYD, was readily soluble in water, but over time, a small amount of gelatinous precipitate was observed. The amount of gelatinous precipitate scales approximately with concentration up to 40 mg/mL. These observations indicate that the propensity of Ac-YYD to aggregate and entrap water is low.

**Figure 2:**
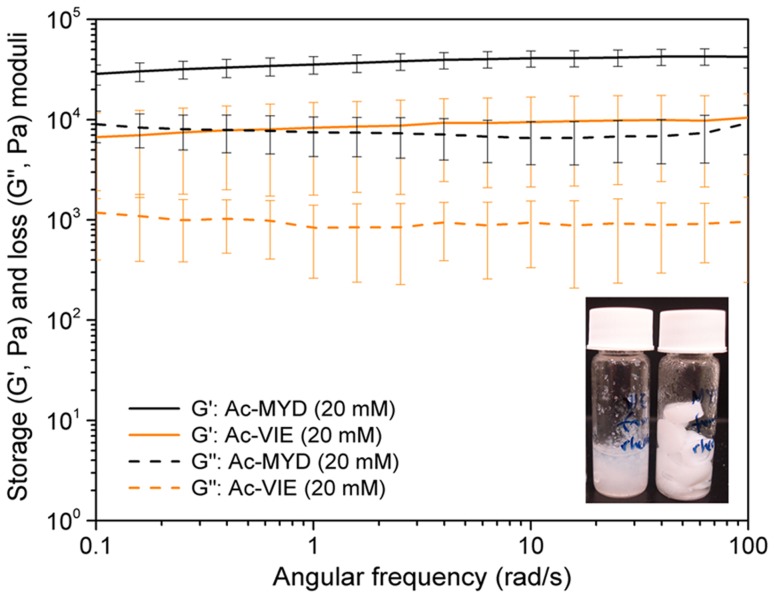
Comparison of the viscoelastic properties of tripeptide. Comparison of the viscoelastic properties of tripeptide hydrogels via frequency sweep studies (strain = 0.1%) at 25°C: Ac-MYD (20 mM, 13 mg/mL), Ac-VIE (20 mM, 12 mg/mL). Every data point represents the mean of 10 repetitions. The error bars reflect the standard deviation of 10 repetitions. The data show that at 20 mM, Ac-MYD formed the stiffer hydrogel compared to Ac-VIE. The inset illustrates the physical forms of the hydrogels (left: Ac-VIE, right: Ac-MYD) on prolonged standing under ambient conditions.

The viscoelasticity of the hydrogels formed from Ac-VIE and Ac-MYD were assessed experimentally at 20 mM. Ac-MYD formed the stiffer hydrogel with a storage modulus (G′) of 20 kPa compared to Ac-VIE (G′ = 8 kPa) (Figure 2). The loss modulus graph also shows that Ac-MYD possessed the larger loss modulus. The loss modulus is a measure of the viscosity of the system, so a substrate with large loss modulus would be very viscous, and less likely to “slip”. Indeed, while the hydrogels of Ac-VIE (G″ = 1 kPa) collapsed within two days, the hydrogel of Ac-MYD (G″ = 9 kPa) was able to maintain its physical form over more than 10 months (Figure 2 inset: note the hydrogel suspended on the wall). The storage modulus values are comparable to those previously reported for the template tripeptide, Ac-IVD [74].

### Experimental Results for Shuffled Control Sequences of Hydrogel Forming Peptides

Control experiments were performed to illustrate the ability of the procedure to compare the relative self-association of analogous tripeptides. Calculations of K^*^_association_ for analogous tripeptides of Ac-MYD and Ac-VIE, based on shuffling the amino acid residues in the tripeptide sequence, were performed. The calculations show that the optimal position of the polar headgroup is at the C-terminal position, which was previously proposed by Hauser et al. [11]. Four tripeptides (Ac-YMD, Ac-DMY, Ac-IVE, Ac-EVI) were chosen from the shuffled sequences and assessed experimentally. As Table 5 shows, the shuffled sequences of Ac-MYD, i.e. Ac-YMD and Ac-DMY, formed clear solutions with no signs of self-association, in agreement with the computed lower K^*^_association_ values (Table 5). While the shuffled sequences of Ac-VIE (i.e. Ac-IVE and Ac-EVI) precipitated with fibrillar nanostructures, hydrogels were not formed (Table 5 and Figure S1). This could be related to the ability of the *de novo* protein design method to predict differently for aliphatic and aromatic tripeptides. It should be noted that the peptides Ac-EVI and Ac-DMY are the only two cases where the self-association motif detailed in Hauser et al. [11] is not incorporated in a tested peptide. The fact that both such peptides are predicted and experimentally validated to not form self-associating structures supports the use of the motif in this and future studies.

**Table 5:** Summary of Approximate Association Affinity (K^*^_association_) values for shuffled sequences of the designed stiff hydrogelating sequences Ac-MYD and Ac-VIE.

Sequence	K^*^_association_	Observation	Sequence	K^*^_association_	Observation
MYD	3.05 E-15	Opaque gel	VIE	5.39 E-64	Translucent hydrogel
YMD	1.23 E-68	Clear solution	IVE	7.60 E-230	Precipitate
DMY	4.91 E -160	Clear solution	EVI	1.40 E-273	Precipitate

### Morphological and Structural Studies of Self-Associating Tripeptides

#### Aliphatic peptides

**Figure 3:**
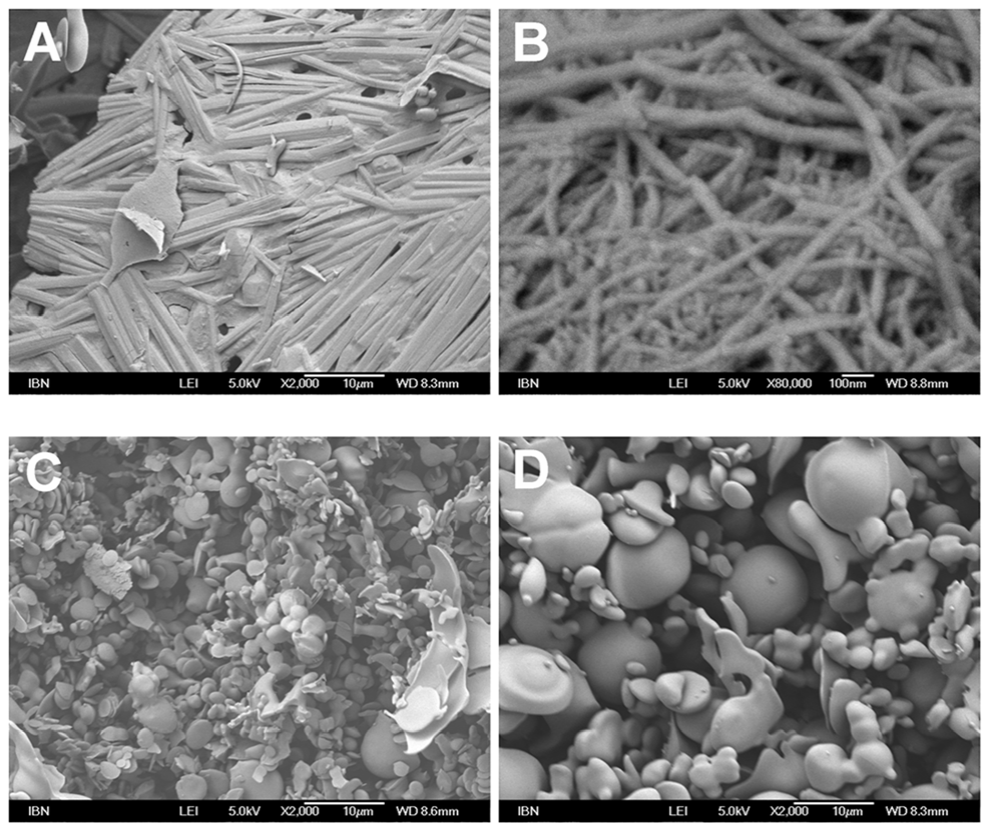
Electron micrographs depicting structures of aliphatic tripeptides after dissolving in water. (A) Ac-LVE; 40 mg/mL, magnification: 2000×, (B) Ac-VIE; 5 mg/mL, magnification: 80000×, (C) Ac-LLE; 5 mg/mL, magnification: 2000×, and (D) Ac-LLE; 40 mg/mL, magnification: 2000×. In the hydrogels of Ac-LVE and Ac-VIE, fibrillar structures were present (thickness: ∼1 µm and ∼30 nm, respectively). In the solutions of Ac-LLE, bead-like structures (diameter: 4–10 µm) were observed at 5 and 40 mg/mL.

To gain information on the morphology that the tripeptides assume on self-association, field emission scanning electron microscopy (FE-SEM) was used to examine the tripeptides after they had been dissolved in water and subsequently freeze-dried. The electron micrographs of the hydrogels of Ac-LVE (40 mg/mL) and Ac-VIE showed the presence of mesh-like fibers (Figure 3A, 3B), the latter being very similar to that observed for Ac-LIVAGD and Ac-IVD [11]. This indicates that Ac-VIE self-assembled into fibers akin to that of Ac-IVD. In contrast, the electron micrographs of Ac-LLE showed no fibrillar features at 5 or 40 mg/mL (Figure 3C, 3D). Instead, bead-like structures (diameter: 4–10 µm) could be observed at 5 and 40 mg/mL (Figure 3C, 3D), as well as at intermediate concentrations (data not shown). This suggests that Ac-LLE associates to form spherical microstructures instead of fibrillar nanostructures.

#### Aromatic peptides

**Figure 4:**
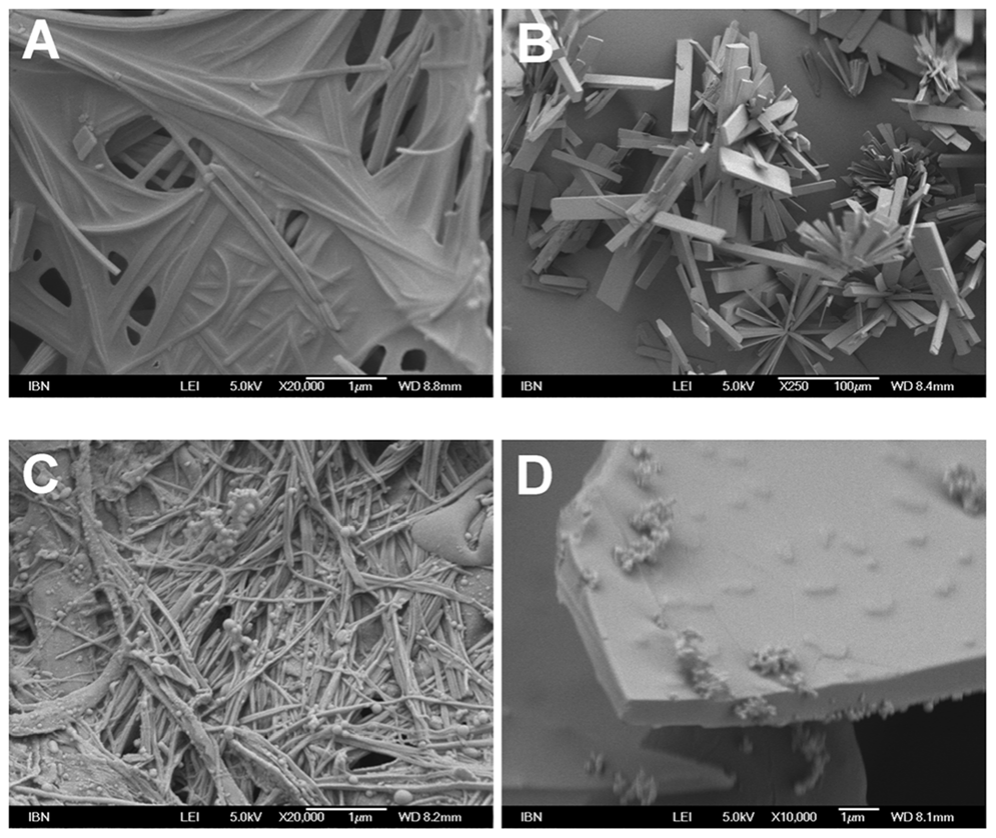
Electron micrographs depicting structures of aromatic tripeptides after dissolving in water. (A) Ac-MYD; 5 mg/mL, magnification: 20000×, (B) Ac-YLD; 40 mg/mL, magnification: 250×, (C) Ac-YYD (precipitate); 40 mg/mL, magnification: 20000×, (D) Ac-YYD (supernatant); 40 mg/mL, magnification: 10000×. In the hydrogel, Ac-MYD formed densely packed fibers (thickness: ∼150 nm). Ac-YLD readily formed large micrometer-sized crystals. In the two phases of Ac-YYD, fibrillar structures (thickness: ∼50 nm) were present in the gelatinous precipitate whereas only amorphous structures were present in the solution.

The electron micrographs of Ac-MYD showed fibers densely packed together (Figure 4A), akin to that observed for various aromatic peptides that have been previously studied [75]. This suggests that it is packed in a different manner to the aliphatic tripeptides. The electron micrographs of Ac-YLD showed the presence of thin rectangular plates with well-defined edges (Figure 4B), as expected of crystals. This reflects the high propensity of Ac-YLD to associate in an orderly manner. Analysis of the gelatinous precipitate of Ac-YYD that had formed slowly showed fibrillar structures (Figure 4C). In contrast to the microstructures observed in the solutions of Ac-LLE, only amorphous structures were observed in the supernatant of Ac-YYD (Figure 4D). This indicates that Ac-YYD possesses the tendency to associate into fibers, but that this tendency is weak.

It should be noted that the use of FE-SEM to visualize nanostructures is not meant to be a direct reflection of the outcomes of the computational simulations, but rather to characterize and compare the nanostructures that can be obtained through the self-association of the designed sequences with the highest calculated tendency to self-associate as quantified by their K^*^_association_ values.

#### Crystallographic study of Ac-YLD

**Figure 5:**
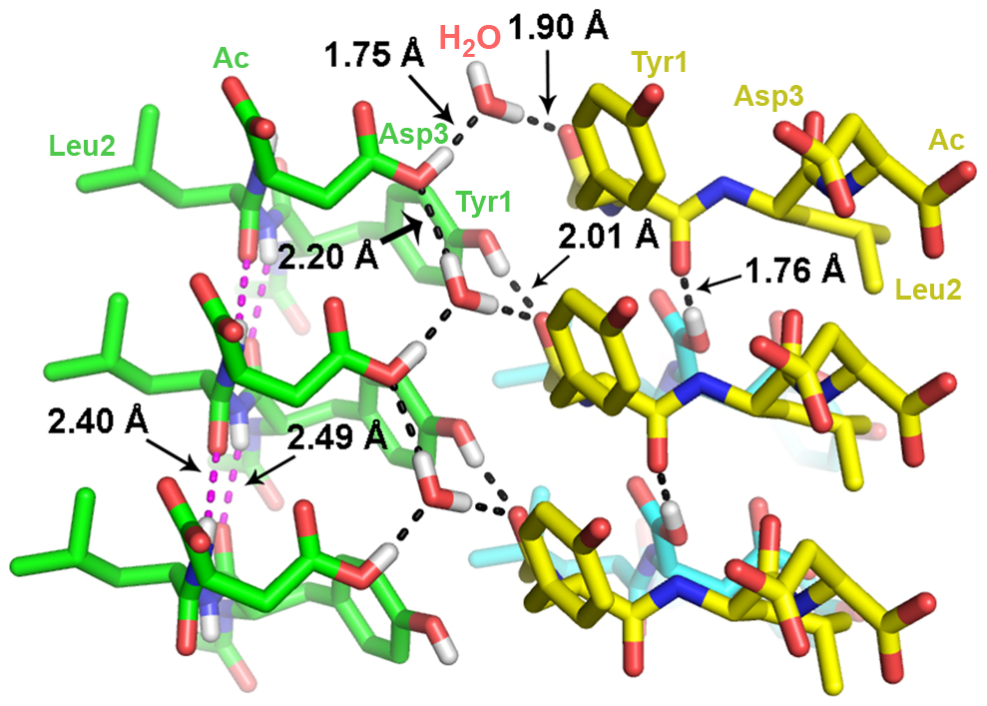
Model of Ac-YLD crystal structure. Stick model illustrating the network of hydrogen bonding linking one molecule of water and three molecules of Ac-YLD together. The intermolecular hydrogen bonds are labeled accordingly. Residue identities are labeled for the first row of peptides only. Most of the hydrogen atoms have been omitted for clarity. The diagram illustrates that the aromatic rings of tyrosine engage in π-π stacking interactions.

An X-ray diffraction study was carried out on a single crystal of Ac-YLD. Ac-YLD adopts the typical conformation of a β-strand, which stacks together to form parallel β-sheets (Figure 5). The β-sheets associate laterally to form the crystal. The side-chain of Leu2 protrudes from one side of the β-sheet while the side-chains of Tyr1 and Asp3 protrude from the opposite side of the β-sheet. A water molecule, anchored by the protonated carboxyl side chain of Asp3, is critical for the formation of a hydrogen bond network that contributes to both intra- and inter-β-sheet interactions. The network is composed of the water molecule and three oxygen atoms (one each) from three adjacent Ac-YLD molecules: Asp3(O-H)-OH_2_, 1.75 Å; Asp3(H-O)-H_2_O, 2.20 Å; Ac(C = O)-H_2_O, 1.90 Å. Other significant hydrogen-bond donor-acceptor pairs include those formed by the protonated C-terminal carboxyl of Asp3 and the amide oxygen of Tyr1 (inter-β-sheet, 1.76 Å), and by the hydroxyl of Tyr1 and the carbonyl oxygen of the N-terminal acetyl (inter-β-sheet, 2.01 Å), as well as two inter-β-strand hydrogen bonds commonly seen in a β-sheet (intra-β-sheet, 2.40 and 2.49 Å, respectively). In addition to these hydrogen bonds, hydrophobic interactions among the clustered hydrophobic moieties, that is, the methyl of the *N*-terminal acetyl and the side-chain of Leu2, also promote both intra- and inter-β-sheet associations. A third type of observed interaction is π-π stacking, which engages the aromatic side chain of Tyr1 in a parallel-displaced manner, and favors the stacking of Ac-YLD into a β-sheet. As Figure 5 illustrates, the 4-phenoyl rings lie “stepped” with respect to each other. The detailed statistics of the crystallization and refinement parameters are shown in Table 6.

**Table 6:** Details of crystallization, data collection and refinement.

**Crystal data**	
Chemical formula	C_21_H_31_N_3_O_9_
*M*_r_	469.49
Crystal system, space group	Monoclinic, *P*2_1_
Temperature (K)	100
*a*, *b*, *c* (Å)	5.0366 (3), 18.0741 (11), 12.6825 (7)
α, β, γ (°)	90, 101.45, 90
*V* (Å^3^)	1131.52 (11)
*Z*	2
Radiation type	Cu Kα radiation, λ = 1.54178 Å
µ (mm^−1^)	0.91
**Data collection**	
Diffractometer	Bruker Kappa APEX-II CCD diffractometer
*θ*_min_, *θ*_max_ (°)	3.6, 56.7
No. of measured, independent and observed [*I*>2σ(*I*)] reflections	3781, 2111, 2071
*R*_int_	0.020
**Refinement**	
*R*[*F* ^2^>2(*F* ^2^)], *wR*(*F* ^2^), *S*	0.025, 0.065, 1.00
No. of reflections	2111
No. of parameters	327
No. of restraints	13
Δρ_max, min_ (e Å^−3^)	0.24, −0.19
Absolute structure	Flack×parameter determined using 581 quotients [(I+)−(I−)]/[(I+)+(I−)] (Parsons et al., 2013)
Absolute structure parameter	0.09 (12)
Absolute structure	Flack×parameter determined using 581 quotients [(I+)−(I−)]/[(I+)+(I−)] (Parsons et al., 2013)
**Computer programs:** *APEX2* (Bruker, 2010), *SAINT* (Bruker, 2010), *SHELXD* (Sheldrick, 2008), *SHELXL2013* (Sheldrick, 2008), *Wincoot* (Emsley, 2010) and *publCIF* (Westrip, 2010).	

The structural data obtained from an X-ray crystallographic study of Ac-YLD, which formed crystals at ambient conditions, has important implications in the further development of the self-association de novo design method. The crystallographic structure could be used as a starting template in future designs in order to increase the accuracy of the Sequence Selection stage and open the possibility of alternate design applications, such as the design of peptides to inhibit crystal formation. Additionally, the structure could be used to gain insight into the physical basis of how Ac-YLD associates to form the crystal which could be used to improve the metrics used in choosing the candidate peptides to more accurately predict which peptides will self-associate into hydrogel or crystal structures.

## Discussion

### Connections between Computational Metrics and Experimental Observations

When evaluating a newly developed multimeric *de novo* peptide design framework that relies on several validation stages, it is important to be able to critically assess each stage separately. The experimental results aim to confirm/disconfirm the predictions that the proposed computational framework makes, thus providing an essential test of the approach. Run 1 utilizes Sequence Selection, Fold Specificity and Approximate Association Affinity to select sequences for experimental validation, whereas Run 2 utilizes only Sequence Selection and Approximate Association Affinity. In order to utilize the framework for reliable prediction of self-associating peptides, it is pertinent to understand the properties that each of Sequence Selection (Pot.E), Fold Specificity (FSpec), and Approximate Association Affinity (K^*^_association_) may influence.

The potential energy used in the Sequence Selection stage, Pot.E, which measures the pairwise interaction energies of residues within the tripeptide, may be indirectly related to the extent to which the tripeptide interacts with the solvent. For instance, if the residues of the tripeptides interact in a highly favorable manner with each other (large negative Pot.E) they may correspondingly interact to a lower extent with the solvent. The converse would also be true. Such substrate interaction with the solvent is known to critically determine the nano-/microstructural form adopted by the substrate. The tripeptides can be grouped into three potential energy classes: low (Ac-LLE; Pot.E = −0.0618), medium (Ac-LVE, Ac-YLD, Ac-YYD; Pot.E = −0.0324, −0.0340, −0.036, respectively), and high (Ac-MYD, Ac-VIE, Ac-IVD; Pot.E = −0.0151, −0.0173, −0.0153, respectively). Ac-LLE (FSpec = 3.54, K^*^_association_ = 4.31×10^−64^) and Ac-YYD (FSpec = 3.89, K^*^_association_ = 2.65×10^−70^) have similar FSpec and K^*^_association_, so the effect of Pot.E on their self-association can be gleaned. With the lower Pot.E, Ac-LLE can interact to a lower extent with water, which may account for the formation of bead-like microstructures. With a higher Pot.E, Ac-YYD can interact to a greater extent with water, which accounts for its high water solubility. The high Pot.E of Ac-MYD/Ac-VIE/Ac-IVD suggests they can interact to the relatively highest extent with water, which accounts for their ability to entrap water in forming hydrogels.

FSpec, which is derived from an ensemble of 500 models with varying backbone conformations, can be construed as sampling conformations that are amenable to self-associating into nano- and microstructures. Indeed, the chosen tripeptides, which all have FSpec more than one, are capable of self-associating into either fibrillar structures (Ac-LVE, Ac-YYD, Ac-MYD, Ac-VIE), crystals (Ac-YLD), or bead-like microstructures (Ac-LLE) to varying extents. This illustrates the capability of the new Fold Specificity metric for multimeric systems.

The Approximate Association Affinity (K^*^_association_) reflects the affinity of the tripeptide to self-associate into multimeric structures. By comparing Ac-LVE (Pot.E = −0.0324, FSpec = 6.09) and Ac-YLD (Pot.E = −0.0340, FSpec = 5.18), which have similar Pot.E and FSpec, the effect of K^*^_association_ on self-association can be assessed. With a greater K^*^_association_, Ac-LVE (1.66×10^−3^) has a higher tendency to self-associate than Ac-YLD (4.97×10^−50^). The higher tendency of Ac-LVE to associate might pre-dispose it to form disorderly aggregates whereas the lower tendency of Ac-YLD to associate could allow it to pack orderly and form crystals. The effect of K^*^_association_ is also borne out by an inspection of the K^*^_association_ of the seven tripeptides: Ac-YYD, which has the smallest K^*^_association_ (2.65×10^−70^) relative to the other six tripeptides, certainly exhibited the lowest affinity to self-associate. Given that Ac-YYD possesses the highest aromatic content of the seven tripeptides, and that aromatic residues are known to self-associate readily via either π-π or CH-π stacking, it is surprising that Ac-YYD would have the lowest tendency to self-associate. Additionally, the negative controls for Ac-VIE and Ac-MYD (i.e., Ac-IVE, Ac-EVI, Ac-YMD, and Ac-DMY) presented in the results demonstrate how the self-association properties of peptides with similar amino acid content can be adequately predicted by the calculated Approximate Association Affinity. These two examples aptly illustrate the capability of the new Approximate Association Affinity metric presented here.

However, it would be remiss to consider that Pot.E, FSpec, and K^*^_association_ independently impact on the self-association outcome of the tripeptides. The group of Ac-MYD/Ac-VIE/Ac-IVD (Pot.E = −0.0151, −0.0173, −0.0153, respectively) provides a case in point. Ac-MYD (K^*^_association_ = 3.05×10^−15^) was observed to possess a higher tendency to gel than Ac-IVD (K^*^_association_ = 4.87×10^−32^), and this could be related to the larger K^*^_association_ of the former. However, although Ac-VIE (K^*^_association_ = 5.39×10^−64^) has a smaller K^*^_association_ than Ac-IVD, it was also observed to gel faster than Ac-IVD. The larger FSpec of Ac-VIE (FSpec = 2.69) compared to Ac-IVD (FSpec = 1.00) suggests that Ac-VIE may adopt conformations that are more amenable to self-association than Ac-IVD, leading to faster gelling. These considerations illustrate how the interplay between FSpec and K^*^_association_ influences the self-association outcome. Naturally, it can be expected that Pot.E would also influence self-association outcome although this is not exemplified in this case. These results demonstrate that both the filtered (Run 1) and unfiltered (Run 2) stages produced experimentally validated tripeptide sequences.

### Interpretation of Single Amino Acid Substitutions

With an interpretation of Pot.E, FSpec, and K^*^_association_, the effects of point mutations in (Ac-LVE↔Ac-LLE) and (Ac-MYD↔Ac-YYD↔Ac-YLD) might be assessed. In all four cases, all three metrics change drastically upon the point mutations. As our results indicate, switching the amino acid from Val to Leu in (Ac-LVE→Ac-LLE) caused the tripeptide to convert from fibrillar structures to bead-like microstructures. Switching the amino acid from the aliphatic methionine (Ac-MYD) to the aromatic tyrosine (Ac-YYD) abolished hydrogelating ability of the tripeptide. This is unlike the aliphatic-to-aromatic residue switch of the amyloid-forming fragment of the human islet polypeptide, in which changing the residue from alanine (NAGAIL) to the native phenylalanine (NFGAIL) led to a gain in amyloid-forming ability [76]. Conversely, switching the amino acid residue from the aromatic tyrosine (Ac-YYD) to the aliphatic leucine (Ac-YLD) led to the facile crystallization of the tripeptide. It is remarkable that such apparently small changes can result in major effects on Pot.E, FSpec, K^*^_association_, and physical properties of the designed peptides. It is tempting to suggest that these changes affect the multimeric structures of the tripeptides, which in turn affect the interaction of the multimeric structures with water [77].

There could be two reasons for the change observed in (Ac-YLD→Ac-YYD): (1) the (4-phenol)methylenyl side-chain of Tyr2 in Ac-YYD would hinder the tight packing of the tripeptide and (2) hydrophobic interactions among the (2-methyl)propyl side-chain of Leu2 in Ac-YLD facilitate the lateral packing of Ac-YLD. Such lateral association of aliphatic side-chains has been noted to be important in the self-assembly of β-hairpin structures that form hydrogels [78]. From the crystal structures of diphenylalanine [79], [80], it can be observed that both intramolecular CH-π interactions [81] and intermolecular π-π stacking [82], [83] are involved in the formation of the nanotubular structure of diphenylalanine. It has often been considered that aromatic groups play a critical role in the key interactions that drive peptide self-assembly, however the extent to which this is true is still unknown [12].

### Comparison of Ac-YLD Crystal Structure to Known PDB Structures

**Figure 6:**
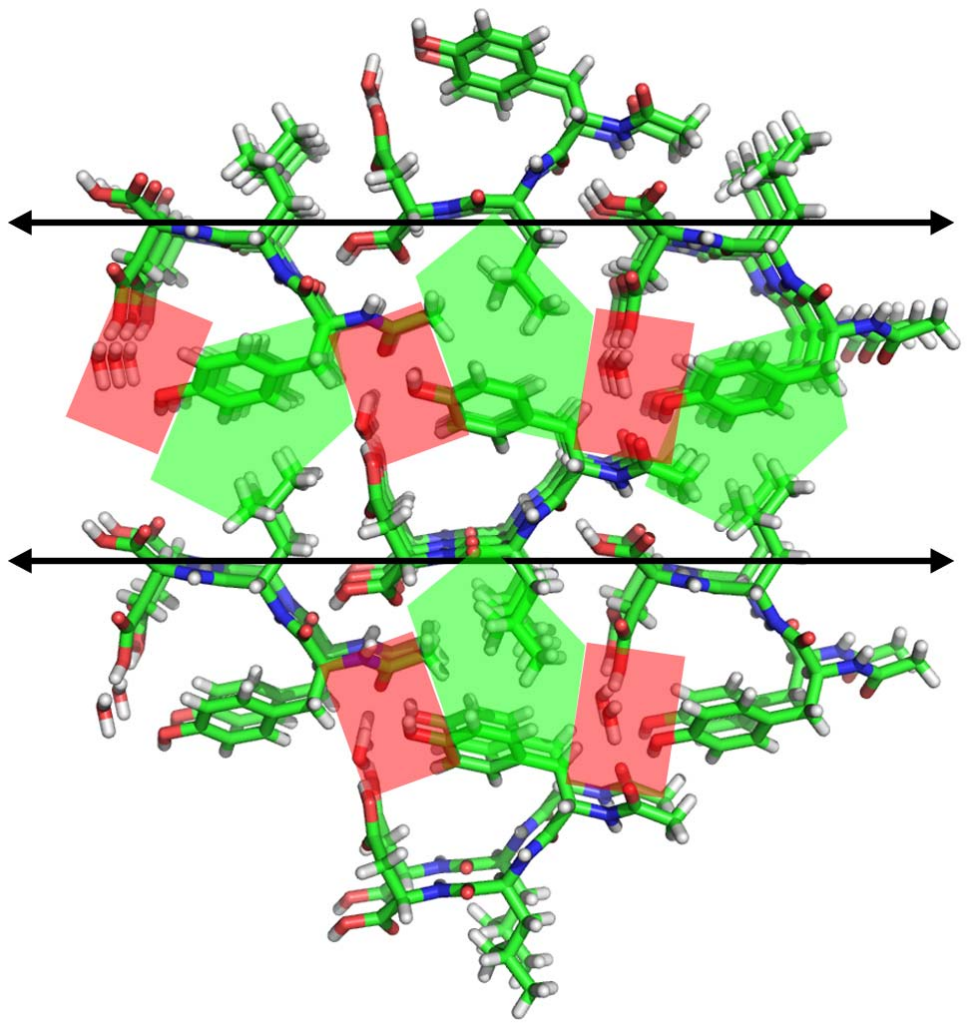
Stick models of the Ac-YLD crystal. Stick model of the Ac-YLD crystal showing a pattern of alternating hydrophobic zipper regions with hydrophilic, water-stabilized regions. Hydrophobic regions are highlighted in green. Water stabilized regions are highlighted in red.

**Figure 7:**
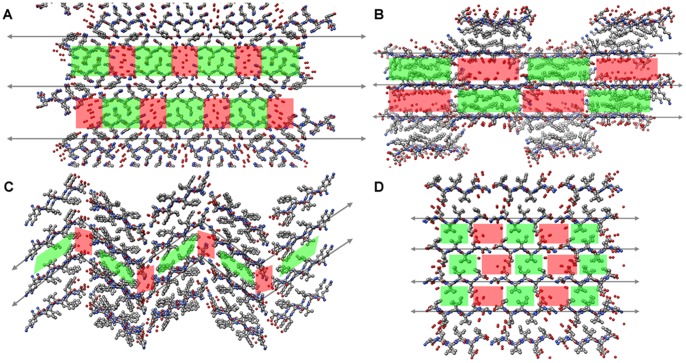
Stick models of small peptide fibril crystals from the PDB. Stick models of small peptide fibril crystals showing a similar pattern to Ac-YLD of alternating hydrophobic zipper regions with hydrophilic, water-stabilized regions. Hydrophobic regions are highlighted in green. Water stabilized regions are highlighted in red. Note that these regions can greatly vary in size and shape across crystal structures. (A) PDB: 2OMM, GNNQQNY (parallel) (B) PDB∶3LOZ, LSFSKD (antiparallel) (C) PDB∶2Y29, KLVFFA (antiparallel) (D) PDB∶3SGS, GDVIEV (parallel).

Analysis of the crystal structure of Ac-YLD in comparison to known crystal structures of small self-associating peptides allows for detailed analysis of the interactions that are important for self-association, and more specifically, those interactions that lead to the formation of ordered crystals. Crystal structures of small, self-associating peptides are rare in the PDB. A total of 96 structures in the PDB are classified as “Protein Fibril”. Of these structures, many have characteristics that make it difficult to compare to the crystal structure of Ac-YLD, such as the presence of modified amino acids, peptide lengths greater than 20 amino acids, presence of stabilizing small molecules, and elucidation by NMR rather than crystallography. Removing structures that contain these characteristics, we are left with 35 PDB structures of associating peptides (Table S3) [9], [10], [84]–[90]. Through analysis of these structures we can identify a consistent motif for crystal stabilization that is also present in the newly determined crystal structure of Ac-YLD. A clear pattern of alternating hydrophobic zipper-like regions and hydrophilic regions stabilized through immobilized water molecules can be found throughout the crystal structure of Ac-YLD (Figure 6). Figure 7A–D provides examples of peptide fibril crystals showing similar patterns, despite their difference in peptide length, sequence, associating properties, and backbone orientation (parallel or antiparallel β-sheet). This suggests that sequences that are amenable to forming such patterns may have a higher tendency for crystal formation. Additionally, the importance of the immobilized water molecule in such peptidic crystals points to the possibility that the inclusion of explicit water molecules in the approximate association energy simulations could improve the prediction of whether a peptide will self-associate into a hydrogel or crystal structure.

### Comparison of Ac-YLD Crystal Structure to Simulation Trajectory

**Figure 8:**
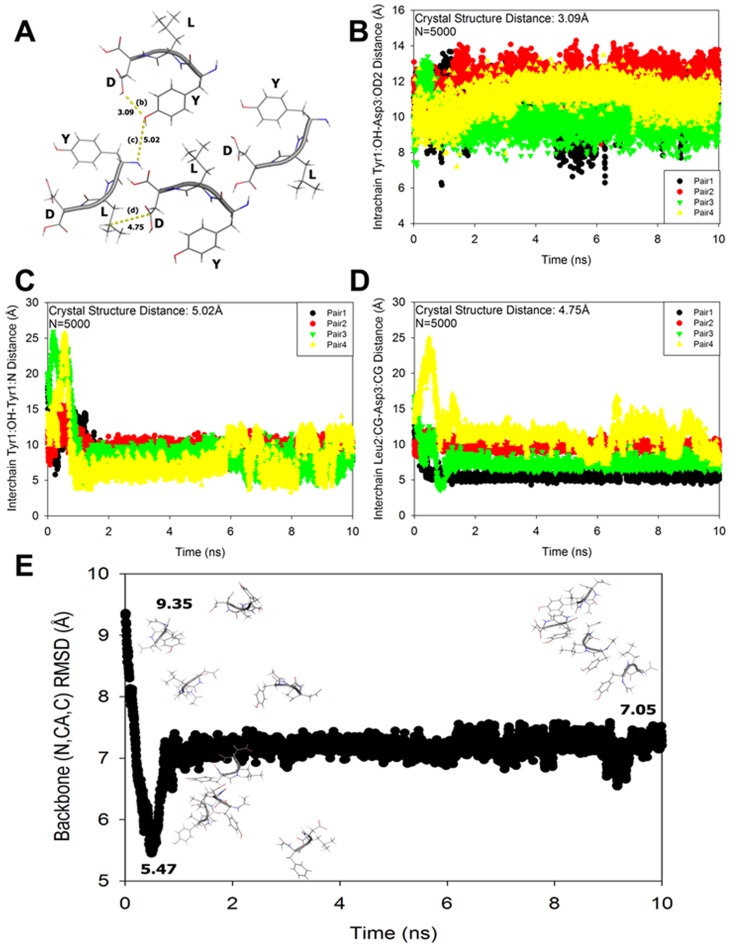
Molecular dynamics simulations vs. crystallography analysis. (A) 4 structures from the periodic structure of Ac-YLD were extracted and key intra and interchain distances were evaluated. They were compared to the distances present in the simulations for (B) intrachain Tyr1∶OH-Asp3∶OD2 (C) interchain Tyr1∶OH-Tyr1∶N and (D) interchain Leu2∶CG-Asp3∶CG. (E) The time-series of the RMSD to the crystal structure of the Ac-YLD is shown with figures representing the initial state, the lowest RMSD state, and the final state in the trajectory. All the states were used for the analysis of the association affinity.

The simulation trajectory of Ac-YLD was compared to the crystal structure of Ac-YLD using VMD [91]. Specifically, key intra- and inter-chain atom distances present in the crystal structure were compared with those sampled in the simulation trajectory. In Figure 8A, one periodic cell consisting of four peptides was extracted from the crystal structure of Ac-YLD. The intra-chain Tyr1∶OH to Asp3∶OD2 distance was 3.09 Å, the inter-chain Tyr1∶OH to Tyr1∶N distance was 5.02 Å, and the inter-chain Leu2∶CG to Asp3∶CG distance was 4.75 Å. Each of these distances were assessed for each of the 5000 frames in the 10 ns trajectory and are shown in Figures 8B, 8C, and 8D, respectively. In the calculation of the inter-chain distances, the corresponding atom on each chain that is closest to the starting chain was used for the calculation. Generally the intra-chain contacts between Tyr1 and Asp3 observed in the crystal structure were not sampled in all of the chains. Conversely, the inter-chain contacts were sampled for a subset of the chains (Figures 8C and 8D). The overall structure at the beginning of the simulation was in a “box-like” configuration with an RMSD to the native of 9.35 Å (Figure 8E). Throughout the simulation trajectory the states sampled became closer to the crystal reaching the minimum distance of 5.47 Å before finding another stable configuration which the multimeric system remained until the end of the simulation at 7.05 Å from the crystal (Figure 8E). The differences in the models sampled and the crystal structure may be due to the initial configuration, or because the models were sampled at a constant temperature. Since we were assessing their strength of interactions, the simulations provide fair comparisons between different sequences of the same length. It is possible that enhanced sampling techniques such as replica-exchange [92] may have allowed for a larger sampling population and should be explored in future work.

While computational de novo design methodologies have advanced in their ability to use simulated structures as input models, as was carried out in this study, it is highly preferable to use experimentally determined structures for design. For this reason the elucidation of a crystal structure for Ac-YLD provides an exciting opportunity for future de novo design studies; in particular, for the potential design of inhibiting peptides that may prevent the observed crystal formation. Designs of this category have biomedical implications for the design of inhibitors of amyloid formation. If the formation of such structures can be prevented by the addition of another small peptide, then the interactions important for such inhibition can be determined and exploited for research into the prevention of the onset of degenerative diseases.

### Conclusions

In this study, we have introduced a new computational *de novo* peptide design framework for multimeric systems and demonstrated its capability to predict self-associating tripeptides based on the metrics of Pot.E, FSpec, and K^*^_association_. Out of the six tripeptides that were computationally predicted to self-associate, all tripeptides formed aggregates of different forms and to different extents, as illustrated by self-association and electron microscopy studies. Two of the six proposed tripeptides, Ac-VIE and Ac-MYD, formed hydrogels at concentrations and on time scales comparable to the template peptide, Ac-IVD. The hydrogel of Ac-MYD showed surprising stability, remaining intact after 10 months, as perhaps might be expected by the computed large association affinity. We were able to use the experimental results to determine how the metrics devised in this work could potentially be used to discriminate between peptides that can and cannot self-associate. Additionally, several negative controls were used to demonstrate the strength of the Approximate Association Affinity metric in distinguishing between closely related peptide sequences that have different self-associating behaviors in nature. These negative controls also support the use of the self-association sequence motif detailed in Hauser et al. [11] as biological constraints in designs of this kind. It is also important to highlight that the aforementioned successful predictions were obtained having as a starting point a simulated initial multimeric structure of IVD and not an experimentally elucidated structure.

Importantly, Ac-YLD produced large crystals at ambient conditions and low concentrations. It is often advantageous to use an experimentally elucidated protein structure as the starting template, rather than a simulated multimeric structure in peptide design. Hence, the Ac-YLD crystal structure can serve as a template basis for the design of additional crystal forming peptides or alternately to design peptidic inhibitors of its crystal formation. The use of a crystal structure as a template in future design will improve the accuracy of the first stage and increase the confidence in the designs produced through the subsequent stages. The crystal structure also provided direct observation of the important interactions for the peptide self-association and common packing features between the crystal structure of Ac-YLD and crystal structures of other small, fibril-forming peptides. It was observed that particular intra-molecular interactions were observed in both the MD simulation and the crystal structure, which may point to which interactions are important for crystal formation and can be used to predict which peptides will form crystals. Furthermore, it was determined that a pattern of alternating hydrophobic and water-stabilized hydrophilic regions exists in many small, peptidic crystals, which may indicate that the inclusion of explicit waters in the simulations may improve the accuracy of the simulations used in the calculation of the Approximate Association Affinity. These types of observations can be used as a guide in refining the de novo design framework which currently has no metric to determine whether gelation or crystal formation takes place.

## Materials and Methods

### Computational Design Methodology

**Figure 9:**
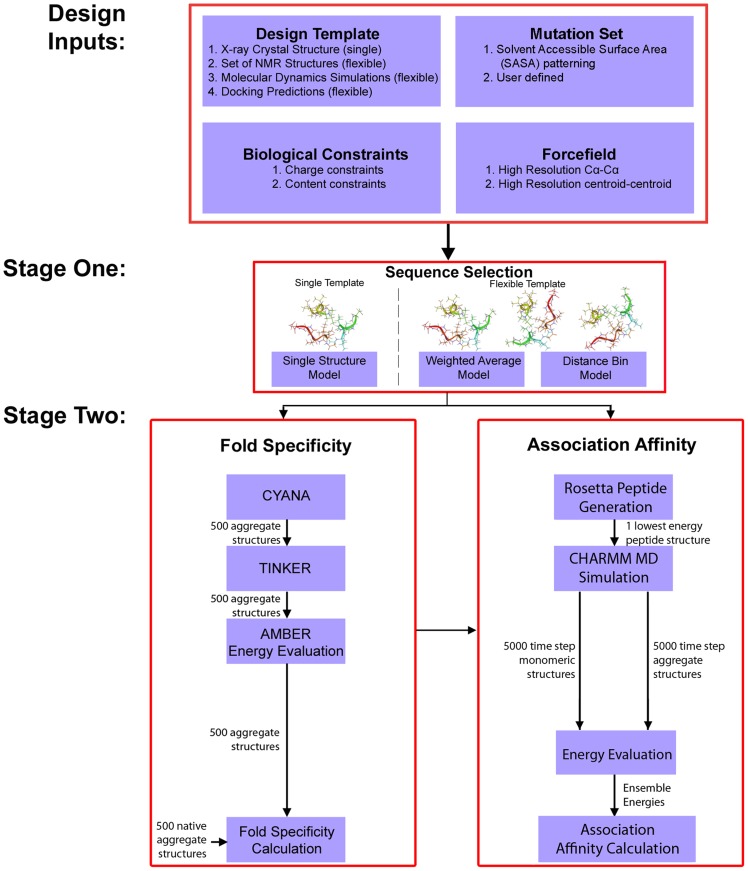
Overview of the de novo protein/peptide design framework for multimeric systems. The method is a two-stage method. Design inputs are used as constraints in an initial optimization sequence selection stage. The sequences identified by the sequence selection stage are then validated computationally by fold specificity and approximate association affinity calculations. High ranking sequences can then be validated experimentally.

The *de novo* protein/peptide design framework applicable to multimeric systems consists of two stages [49], [50], [52], [53], [59], [70]–[72]. The framework has been developed to handle flexible backbone templates, since experimental structures are not often available for multimeric systems. As such, a flexible backbone template must be created through simulation. In the current design of self-associating tripeptides, MD simulations were performed for this purpose, which produced many snapshots of the plausible multimeric complex. These snapshots were then used to produce a flexible backbone template. The flexible backbone template was subsequently used as the input for the design framework. The first stage of the framework is Sequence Selection, which is based on a global optimization method that minimizes the potential energy of a designed sequence in the flexible template structure. The potential energy used can either be based on an 8-bin Cα-Cα force field or an 8-bin centroid-centroid force field [93], [94]. A novel aspect of this method is the mathematical connection of residues in the design framework, so that identical chains in the template structure remain identical throughout the design procedure. The optimized sequences are then subjected to a Fold Specificity calculation and screening. Fold Specificity assesses how energetically favorable it is for the designed sequence to adopt the target multimeric structure in comparison to the native sequence. In cases where the native sequence is known to associate, Fold Specificity aims to produce designed sequences that are more energetically favorable in the target multimeric structure than the native sequence. In cases where the native sequence does not assemble, sequences with higher Fold Specificity are considered to have a higher chance of adopting the novel multimeric structure. Finally, a subset of high confidence sequences is subjected to an additional validation step whereby MD simulations are used to dynamically assess the energetics of each designed sequence and its potential to self-associate. In this type of design problem, the binding of several peptides into a multimeric structure has to be considered, which is tackled by the novel Association Affinity metric. All the steps in the design framework, which are presented in a workflow diagram (Figure 9), are defined in full detail in the following section. This framework is a general methodology that can be applied to a variety of multimeric protein/peptide design problems.

### Design Template

PyRosetta [95] was used to generate the initial tripeptide models for the template sequence Ac-IVD through a Monte Carlo (MC) conformational search. The function “make_pose_from_sequence” was used in conjunction with the “fa_standard” Rosetta force field [96]. A SmallMover object was constructed with the backbone being allowed to move, with 5 MC perturbations per cycle. The model was subjected to 60,000 MC cycles, with the Metropolis criterion determining whether a move was accepted or rejected. This procedure was used to generate 200 low energy decoys for the template. The models were then clustered in Rosetta. The four lowest energy models from the densest cluster were centered at the origin.

In CHARMM, the four tripeptides were translated 8 Å in both the y- and z- directions so as to form a square box with the distance from the center of the box to the center of each peptide being 11.31 Å. Each tripeptide was rotated randomly. “Hbuild” was used to construct the hydrogen atoms. Periodic boundary conditions, which determine the length and (consequently) volume of the box, were applied in CHARMM so that the concentration of the system was 20 mg/mL. The nonbonded cutoffs in CHARMM were set using the following options and values: ctonnb 20, ctofnb 20, cutnb 24, and cutim 24. Implicit solvent was invoked using the generalized Born with simple switching model [97]. A half smoothing length of 0.3 Å, a non-polar surface tension coefficient of 0.03 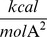, and a grid spacing of 1.5 Å were used. The system was subjected to 2,000 steps of steepest descent, followed by 2,000 steps of adopted basis-set Newton-Raphson, and finally an additional 2,000 steps of steepest descent minimization. The system was heated to 300 K over 10 ps (stepsize: 0.5 fs) and with harmonic constraints on all heavy atoms with force constant 5.0 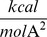. The system (N,V,T ensemble) was equilibrated for 1 ns (stepsize: 1 fs) with a force constant of 1.0 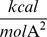 on all heavy atoms. The system was subjected to 10 ns of molecular dynamics (MD) at 300 K, with SHAKE constraints applied to all bonds involving a hydrogen atom with a tolerance of 1–10. All simulations were performed using Langevin dynamics with the leapfrog integration scheme. The last 5 ns of the simulation trajectory were processed into pdb files, which were used as the flexible template for design.

### Biological Constraints, Mutation Set, and Force Field

In accordance to the amphiphilic profile of the template sequence Ac-IVD, the motif [hydrophobic]-[hydrophobic]-[E/D] was applied in the computational method. The hydrophobic residues were allowed to mutate to Leu, Ile, Val, Ala as utilized in the self-assembling hexapeptide Ac-LIVAGD [11], as well as Met, Phe, Tyr, and Trp. Aromatic moieties have been observed to be important for association due to π-stacking, so aromatic residues were included to expand the scope of tripeptides available for comparison. This resulted in a total of 128 candidate tripeptide sequences, which is a small enough pool that no further biological or mutational constraints were required. Previously developed 8-bin Cα-Cα and centroid-centroid force fields [93], [94] were available for use as the potential energy function in the Sequence Selection stage. In this study, the 8-bin centroid-centroid force field was employed, since unlike the Cα-Cα force fields, the centroid-centroid force field implicitly includes side-chain directionality in the potential energy calculation.

### Sequence Selection

Since the template for tripeptide association was determined through MD simulations, the optimization-based Sequence Selection method was used with a flexible template rather than a rigid backbone template. Two previous methods were developed for flexible template protein design: a Weighted-average method and a Distance Bin method [70]–[72]. The Weighted-average method takes into account each flexible template, such that the potential energy of the system is the average of all the determined templates in the flexible ensemble. The Distance Bin method allows for the optimization method to design not only for the sequence, but also for the optimal interaction distances for each residue-residue interaction. The Distance Bin method represents the most rigorous way of designing with a flexible backbone. For this reason, the Distance Bin Sequence Selection framework for multimeric system design was the chosen framework that was used in this study.
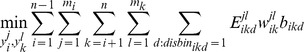
Subject to
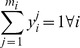


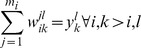


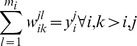


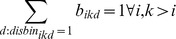


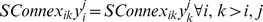


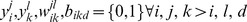
(1)


The model minimizes the summation of pairwise interaction energies 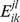, which is the interaction between residue types *j* and *l* in residue positions *i* and *k* whose distance apart falls in distance bin *d*. The binary variable 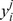 equals 1 if residue type *j* is in residue position *i*, and 0 otherwise. The binary variable 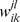 equals 1 if, and only if, 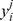 and 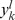 are both equal to 1, and is 0 otherwise. This represents an exact linearization of the problem. The final binary variable 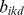 is allowed to equal 1, if and only if, the distance between positions *i* and *k* fall into distance bin *d* in at least one of the flexible models in the template. In this way, the model is allowed to select one, and only one, distance bin in which two residues can fall, from the set of distance bins observed in the flexible template. A new element of this model is the addition of a mathematical parameter denoted here as 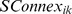 to connect design constraints between multiple chains. This parameter is defined as 1 if two design positions (*i* and *k*) are identical positions in a design system. For example, in the design of a dimer, two identical positions in the two proteins will not allow the model to design for one of the positions without designing for the other position as well. It is also important to emphasize that the objective function is a pairwise interaction potential energy, which takes into account the possible structural flexibility and mutational constraints through a series of linear constraints. The minimization of this objective function aims to improve the stability of the designed sequences in the target structure. This model was used to energetically evaluate all possible tripeptide sequences that fit to the defined design motif. This constituted a total of 128 possible designed sequences, a small enough pool to allow for an exhaustive design search and validation for each sequence. This provides an ideal test system for the new design method as all possible design sequences could be evaluated at each stage before experimental validation.

### Fold Specificity

To further validate and analyze the 128 possible tripeptides, a method capable of calculating the Fold Specificity [70] for sequences in multimeric systems was developed. This method uses a constrained annealing simulation in CYANA [98], [99] to produce a set of initial models. A local AMBER energy minimization using TINKER [100] is then performed on each model to produce a set of 500 final models, along with corresponding AMBER ff94 energy values [101]. Using these AMBER energy values the Fold Specificity value is calculated as follows:
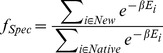
(2)where “New” is the set of models produced for the new sequence, “native” is the set of models produced for the reference sequence Ac-IVD, and 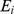 is the AMBER energy value calculated for model *i*. Physically, Fold Specificity assesses how energetically favorable it is for the designed sequences to adopt the target multimeric structure in comparison to the native sequence. The aim is to assess the specificity of the designed sequences for the target structure using a more detailed, atomistic potential energy than in the Sequence Selection stage. Since the metric compares the energy values of the designed sequence directly to those of the native sequence, a “favorable” sequence is one that has a Fold Specificity value greater than 1.

### Approximate Association Affinity Calculation

PyRosetta was used to construct initial coordinates of the subset of the 128 tripeptide sequences that ranked highly in the Fold Specificity metric. The MC and MD protocols, which were described previously in the Template Generation section, were used to generate a trajectory for each candidate sequence. The ensemble of models generated through this dynamics run could then be used in the calculation of an Approximate Association Affinity of four tripeptides associating together in the simulations. Since the tripeptides have high flexibility, they do not have a single stable state. Thus, the simulations did not attempt to reproduce the three-dimensional structure of an associate precursor [11], but to provide an estimation of the affinity of a particular sequence to itself through physics-based intermolecular interactions.

For the equilibrium association of two species A and B in solution, the binding affinity can be calculated as:
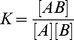
(3)Lilien et al. [73] proposed an approach for the calculation of approximate binding affinities of protein-ligand complexes. It was based on generating rotamerically-based ensembles of the protein, ligand, and protein-ligand complex. These ensembles were used then to calculate partition functions. This approximate binding affinity was denoted as 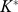 and defined by Eq. 4:
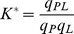
(4)

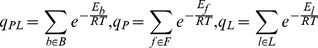
(5)where 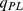 is the partition function of the protein-ligand complex, 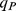 is the partition function of the free protein, and 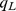 is the partition function of the free ligand. The partition functions are defined in Eq. 5, where the sets *B*, *F*, and *L* contain the rotamerically-based conformations of the bound protein-ligand complex, free protein, and free ligand, respectively. The value 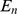 is the energy of conformation *n*, *R* is the gas constant, and *T* is the temperature.

A similar metric can be defined for the association of 4 monomeric peptides into a homogeneous multimeric system. This metric, referred to as the Approximate Association Affinity, is defined as:
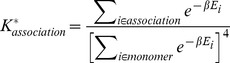
(6)This metric was used in conjunction with the Jacobi logarithm [102] to avoid numerical overflow in the calculation. K^*^_association_ was calculated for each candidate sequence and rank-ordered from the highest (most favorable spontaneous association) to the lowest (Table S1). The simulation of each design was then visually inspected to assess whether the tripeptides associated during the simulation and thus fit to the model. Sequences that did not associate were not considered regardless of the value of the metric. The final set of designed sequences picked for experimental assessment was selected via a combination of Potential Energy, Fold Specificity, and Approximate Association Affinity. The criteria for this selection are provided in more detail in the Results.

### Experimental Methods

#### Materials and preparations

The tripeptides (Ac-LVE, Ac-YYD, Ac-LLE, Ac-YLD, Ac-MYD, Ac-VIE) were purchased from American Peptide Company (Sunnyvale, CA, USA), while Ac-YMD, Ac-DMY, Ac-IVE, and Ac-EVI were manually synthesized via solid phase peptide synthesis [103] and purified to >95% via HPLC. Amino acid content (AA %) analysis was performed and the actual peptide content of each sample was determined by calculating the net weight (gross weight×AA %). The peptides were dissolved by vortexing in deionized water. The tripeptide samples were prepared from 5 to 40 mg/mL, in steps of 5 mg/mL, to assess and compare the association properties of the tripeptides. To allow for proper self-assembly, all samples were left untouched for 24 h before further analyses.

#### Field emission scanning electron microscopy studies

The peptide samples were flash-frozen at −80°C and subsequently freeze-dried. The dried samples were adhered onto copper conductive tape on a sample holder and sputtered with platinum for 60 seconds in a JEOL JFC-1600 High Resolution Sputter Coater operating at a coating current of 20 mA. Examination of sample morphology was carried out on a JEOL JSM-7400F Field Emission Scanning Electron Microscopy system with an accelerating voltage of 5 kV and emission current of 10 mA. Electron micrographs were acquired in the lower secondary electron imaging (LEI) mode using a working distance of 8–9 mm.

#### Rheological measurements

The peptide solutions (20 mM, 250 µL) were pipetted into a polystyrene ring mold as described previously [104]. The solutions were allowed to gel at ambient temperature (22°C) over 24 hours before rheological measurements were carried out. Ten hydrogel samples of each peptide candidate were prepared and measured. The viscoelastic properties of the hydrogels were measured at 25°C on an ARES-G2 rheometer (TA Instruments, Piscataway, NJ) equipped with an 8.0 mm-diameter titanium serrated plate. Upon loading the hydrogel on the rheometer platform and adjusting the height of the measurement plate, the system was allowed to equilibrate for 300 s. The oscillatory frequency sweep study was performed across 0.1–100 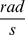 at a constant strain of 0.1%, which was followed by an equilibration period of 300 s and subsequently, an amplitude sweep study performed across 0.1–100% at a constant oscillatory frequency of 6.6 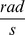.

#### X-ray crystallography studies

Ac-YLD in a glass vial was dissolved in water to 5 mg/mL, and was allowed to crystallize spontaneously at ambient temperature for 24 h. The crystals were then transferred into 25% (v/v) glycerol for 5 min before being flash frozen in liquid nitrogen. X-ray diffraction data was collected at −173°C on a Bruker X8 PROTEUM system consisting of a MICROSTAR micro-focus X-ray generator, a PLATINUM135 CCD detector, and a 4-circle KAPPA goniometer. Data reduction was carried out using SAINT, SADABS, and XPREP, which are part of the Bruker PROTEUM2 program suite (Bruker AXS inc.) [105]. *Ab initio* structural solution was achieved using SHELXD [106], and the model obtained was further refined using SHELXL [107] (through the ShelXle graphical user interface [108]). Details of crystallization, data collection and refinement are listed in Table 5. The final structure was deposited at the Cambridge Crystallographic Data Centre with the deposition numbers CCDC 974865 (Data S1 and Data S2).

## Supporting Information

Figure S1 **Pictures of peptides (shuffled sequences) in water.** Row 1: (From left to right) Ac-YMD and Ac-DMY in water at various concentrations. Row 2: (From left to right) Ac-IVE and Ac-EVI in water at various concentrations.
(PDF)

Table S1 **Full Stage II computational validation results.** All 109 peptide sequences tested for Fold Specificity and Approximate Association Affinity are provided. The table is ordered by Approximate Association Affinity as this was the metric used in selecting the final experimentally validated peptide sequences. Additionally, average and median interaction energies of the self-associating peptides were calculated and are provided.
(PDF)

Table S2 **Full Stage I sequence selection results.** Sequence Selection Potential Energy results were calculated for all 128 peptides possible given the design constraints and are provided here. The table is ordered by Potential Energy.
(PDF)

Table S3 **PDB structures and references for self-associating peptides.** All 35 comparable PDB structures of self-associating peptides are reference. A subset of these peptides were used to compare to the crystal structure of Ac-YLD.
(PDF)

Data S1 **YLD.pdb: X-ray crystallography structure of Ac-YLD in PDB format.** The x-ray crystallography structure of Ac-YLD is provided in PDB format. This file can be viewed in programs such as Chimera, PyMOL, Jmol, or VMD.
(PDB)

Data S2 **YLD.cif: X-ray crystallography structure of Ac-YLD in CIF format.** The x-ray crystallography structure of Ac-YLD is provided in Crystallographic Information File (CIF) format. This file can be viewed in programs such as enCIFer, Jmol, or RasMol. The final structure was deposited at the Cambridge Crystallographic Data Centre with the deposition number CCDC 974865.
(CIF)
