# Supplementary material for: De Novo Design and Experimental Characterization of Ultrashort Self-Associating Peptides
Source: PLoS Comput Biol. 2014 Jul 10;10(7):e1003718. doi: 10.1371/journal.pcbi.1003718 (PMC4091692; doi:10.1371/journal.pcbi.1003718)

Figure S1: Pictures of Peptides (Shuffled Sequences) in Water. Row 1: (From left to right) Ac-YMD and Ac-DMY in water at various concentrations. Row 2: (From left to right) Ac-IVE and Ac-EVI in water at various concentrations.

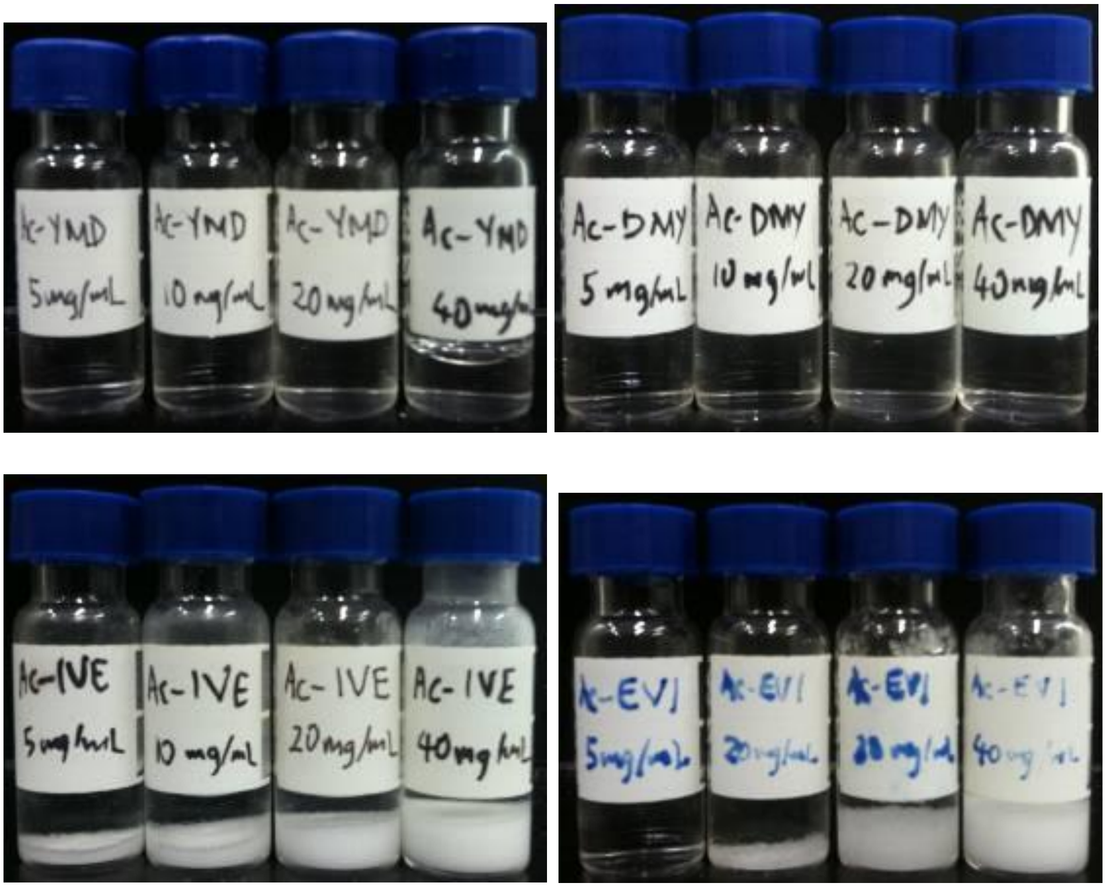

Supplement: Figure S1 — Pictures of peptides (shuffled sequences) in water. Row 1: (From left to right) Ac-YMD and Ac-DMY in water at various concentrations. Row 2: (From left to right) Ac-IVE and Ac-EVI in water at various concentrations. (PDF) [file pcbi.1003718.s001.pdf]
