# Supplementary material for: De Novo Design and Experimental Characterization of Ultrashort Self-Associating Peptides
Source: PLoS Comput Biol. 2014 Jul 10;10(7):e1003718. doi: 10.1371/journal.pcbi.1003718 (PMC4091692; doi:10.1371/journal.pcbi.1003718)
Supplement: Table S1 — Full Stage II computational validation results. All 109 peptide sequences tested for Fold Specificity and Approximate Association Affinity are provided. The table is ordered by Approximate Association Affinity as this was the metric used in selecting the final experimentally validated peptide sequences. Additionally, average and median interaction energies of the self-associating peptides were calculated and are provided. (PDF) [file pcbi.1003718.s002.pdf]

Table S1: Full Stage II Computational Validation Results. All 109 peptide sequences tested for Fold Specificity and Approximate Association Affinity are provided. The table is ordered by Approximate Association Affinity as this was the metric used in selecting the final experimentally validated peptide sequences. Additionally, average and median interaction energies of the self-associating peptides were calculated and are provided.

K* Rank	P1	P2	P3	Stage I Energy	Fold Spec.	<Inter. Energy>	K*assoc	<Etotal>	Median IE
1	LEU	VAL	GLU	-0.03	6.07	5.79	1.66E-03	460.27	4.38
2	MET	TYR	ASP	-0.02	1.37	-11.53	3.05E-15	411.46	-13.04
3	ALA	ALA	ASP	-0.01	0.71	-0.19	1.33E-24	-94.75	-0.21
4	ILE	TYR	GLU	-0.03	0.84	-14.93	3.97E-30	135.72	-15.75
5	ILE	TYR	ASP	-0.03	0.43	-8.86	2.23E-30	193.52	-9.58
6	ILE	VAL	ASP	-0.02	1.00	14.14	4.87E-32	-149.97	15.06
7	TRP	VAL	ASP	-0.05	0.65	4.46	7.80E-33	-23.86	2.24
8	LEU	ALA	ASP	-0.03	0.44	3.72	1.04E-33	560.80	2.81
9	ALA	TRP	ASP	-0.05	0.18	-13.27	8.25E-36	439.36	-14.29
10	TRP	TRP	ASP	-0.13	0.06	-7.76	2.15E-36	291.94	-8.26
11	PHE	MET	ASP	-0.03	0.28	-7.52	2.52E-39	698.21	-7.64
12	TYR	MET	GLU	-0.01	1.32	-19.50	2.34E-40	303.97	-20.45
13	ALA	ILE	ASP	0.00	0.23	2.85	6.70E-42	166.13	1.74
14	TYR	ALA	ASP	-0.03	0.77	-17.57	4.48E-43	-129.41	-19.30
15	PHE	VAL	GLU	-0.03	1.08	-2.22	1.09E-43	577.84	-4.43
16	MET	TRP	ASP	-0.05	0.07	-16.25	4.34E-46	264.48	-18.20
17	MET	LEU	ASP	-0.03	1.62	-3.87	1.10E-46	347.93	-5.03
18	PHE	TRP	GLU	-0.05	0.26	-13.34	1.72E-47	741.30	-14.29
19	ALA	PHE	ASP	-0.03	0.18	3.34	4.62E-48	678.21	1.23
20	TYR	ALA	GLU	-0.03	1.10	4.33	5.32E-49	249.28	-12.23
21	ALA	TYR	ASP	-0.03	0.60	14.02	2.42E-49	299.98	0.43
22	ILE	MET	ASP	-0.02	0.30	4.98	2.20E-49	160.36	3.97
23	TYR	LEU	ASP	-0.03	5.18	-0.49	4.97E-50	207.42	-1.18
24	TYR	ILE	ASP	-0.03	0.91	-4.92	4.97E-50	407.60	-8.24
25	LEU	VAL	ASP	-0.02	1.71	2.72	5.56E-51	278.43	2.02
26	TYR	TRP	ASP	-0.08	0.27	-1.35	3.70E-51	751.18	-3.95
27	TRP	ALA	ASP	-0.05	0.08	-6.19	4.34E-52	382.29	-7.14
28	VAL	PHE	ASP	-0.03	1.22	-3.31	7.35E-54	193.40	-3.69
29	VAL	LEU	GLU	-0.03	5.17	3.88	1.12E-54	58.50	3.45
30	VAL	ALA	GLU	-0.01	2.07	7.25	1.41E-57	589.24	6.32
31	MET	MET	ASP	0.01	0.52	-8.64	1.11E-57	772.15	-9.53
32	TRP	LEU	ASP	-0.07	0.28	7.96	4.19E-58	-8.61	7.32
33	LEU	TRP	ASP	-0.07	0.54	2.11	2.09E-58	147.44	2.01
34	VAL	ILE	ASP	-0.02	0.67	0.39	1.89E-60	620.14	-0.65
35	TYR	PHE	ASP	-0.05	0.96	17.69	3.92E-61	-71.81	17.33
36	TYR	ILE	GLU	-0.03	2.66	1.75	1.34E-61	377.49	-5.03
37	ALA	TYR	GLU	-0.03	2.04	-5.21	5.86E-62	157.82	-5.67
38	PHE	VAL	ASP	-0.02	0.53	9.25	5.53E-62	561.18	9.09
39	TRP	MET	ASP	-0.06	0.19	-0.16	3.72E-62	718.27	-3.28

40	LEU	ILE	ASP	-0.04	0.87	16.85	9.83E-64	197.01	19.10
41	VAL	ILE	GLU	-0.02	2.69	10.10	5.39E-64	366.76	6.89
42	LEU	LEU	GLU	-0.06	3.54	-6.10	4.31E-64	34.78	-6.72
43	ALA	PHE	GLU	-0.02	0.55	-0.23	7.92E-66	410.02	-0.72
44	ALA	ILE	GLU	0.00	0.50	14.67	5.81E-66	238.33	8.00
45	MET	VAL	GLU	0.00	1.61	0.39	4.99E-67	294.28	-0.27
46	TRP	PHE	ASP	-0.06	0.12	21.79	4.03E-67	265.87	18.36
47	TYR	MET	ASP	-0.02	1.67	-3.79	1.23E-68	337.02	-4.36
48	PHE	TYR	GLU	-0.05	0.88	18.54	9.91E-69	775.34	11.36
49	PHE	LEU	ASP	-0.03	0.92	0.73	7.28E-70	569.67	0.21
50	TYR	TYR	ASP	-0.04	3.89	38.53	2.65E-70	141.08	16.84
51	LEU	TYR	ASP	-0.04	2.39	7.05	2.67E-72	279.82	-0.58
52	LEU	MET	GLU	-0.03	1.11	3.78	2.53E-72	203.02	3.36
53	MET	LEU	GLU	-0.03	1.80	50.14	1.72E-72	566.74	18.92
54	ALA	MET	GLU	0.00	1.18	-5.88	1.42E-72	311.42	-6.44
55	ALA	TRP	GLU	-0.04	0.16	-3.24	1.03E-73	299.62	-3.55
56	LEU	LEU	ASP	-0.05	1.75	0.12	8.26E-75	543.62	-0.27
57	VAL	TYR	ASP	-0.02	2.11	-10.54	1.85E-75	540.31	-11.57
58	TYR	TRP	GLU	-0.08	1.07	-3.39	3.98E-76	497.25	-7.32
59	MET	ALA	GLU	0.00	0.58	6.58	1.29E-76	430.81	5.30
60	ALA	LEU	ASP	-0.02	1.71	13.07	1.16E-76	231.68	10.68
61	TRP	TYR	GLU	-0.09	0.45	-10.41	1.47E-78	153.81	-11.81
62	TYR	LEU	GLU	-0.05	8.48	11.82	1.13E-79	106.98	7.61
63	TRP	PHE	GLU	-0.06	0.27	-7.77	6.89E-81	711.63	-9.32
64	ILE	LEU	GLU	-0.04	1.05	6.18	1.42E-81	366.31	5.63
65	LEU	PHE	GLU	-0.04	1.29	-0.15	5.85E-84	379.35	-1.40
66	PHE	PHE	GLU	-0.01	0.45	-6.93	1.87E-88	292.37	-9.71
67	MET	PHE	GLU	-0.02	0.23	104.65	1.40E-88	178.23	43.12
68	VAL	TRP	ASP	-0.05	0.38	-2.29	7.72E-90	610.83	-2.33
69	VAL	MET	GLU	-0.01	3.02	13.32	4.23E-90	325.35	12.30
70	MET	ALA	ASP	-0.01	0.41	10.20	2.40E-93	542.62	11.14
71	PHE	ILE	GLU	-0.02	0.58	3.03	1.38E-93	374.70	2.83
72	MET	VAL	ASP	-0.01	1.43	-0.25	2.12E-94	526.58	-1.08
73	VAL	TRP	GLU	-0.05	0.68	-8.80	6.92E-96	319.17	-8.83
74	LEU	PHE	ASP	-0.04	0.62	1.75	4.70E-98	572.49	0.99
75	MET	ILE	GLU	0.00	0.91	9.01	5.36E-101	322.88	8.34
76	LEU	ILE	GLU	-0.04	0.85	4.62	1.71E-104	371.53	5.51
77	TYR	PHE	GLU	-0.06	1.66	3.01	2.59E-107	246.61	2.07
78	LEU	MET	ASP	-0.03	1.14	-4.11	5.33E-108	501.87	-5.05
79	ILE	MET	GLU	0.00	0.28	3.22	1.14E-110	433.22	1.87
80	LEU	ALA	GLU	-0.03	1.07	5.35	9.10E-119	363.24	3.64
81	TRP	LEU	GLU	-0.08	1.12	2.10	1.16E-119	898.88	-0.51
82	PHE	MET	GLU	-0.02	0.26	7.91	4.14E-120	741.09	-2.64
83	PHE	ALA	GLU	-0.02	0.28	3.90	9.41E-122	490.49	3.28
84	TYR	VAL	GLU	-0.03	6.96	-3.72	7.25E-124	578.86	-5.12

85	ILE	ILE	GLU	-0.02	0.33	9.90	1.27E-128	467.58	7.24
86	VAL	VAL	ASP	-0.01	5.73	87.16	7.72E-137	-1959.52	22.66
87	ILE	PHE	ASP	-0.03	0.12	-0.12	1.45E-138	393.34	0.84
88	LEU	TRP	GLU	-0.07	0.42	-9.20	8.78E-149	408.65	-10.43
89	PHE	ALA	ASP	-0.03	0.32	1.56	1.55E-149	718.21	1.00
90	VAL	ALA	ASP	-0.01	1.24	8.34	7.88E-153	387.58	4.83
91	TYR	TYR	GLU	-0.05	5.79	-25.45	2.81E-161	251.52	-27.49
92	TRP	VAL	GLU	-0.05	0.52	-8.19	3.96E-165	574.29	-9.77
93	ILE	LEU	ASP	-0.03	0.92	7.63	5.60E-171	403.06	6.64
94	PHE	LEU	GLU	-0.04	0.84	30.10	1.62E-179	497.89	24.13
95	ILE	ALA	GLU	0.00	0.28	11.62	4.09E-181	362.34	11.25
96	MET	ILE	ASP	-0.02	0.25	-9.21	4.94E-182	878.14	-10.05
97	ALA	ALA	GLU	-0.01	0.66	1.80	9.44E-183	467.33	0.69
98	ALA	VAL	ASP	-0.01	1.75	1.21	2.94E-186	367.28	0.15
99	MET	MET	GLU	0.03	0.91	-8.64	1.87E-191	715.16	-9.53
100	LEU	TYR	GLU	-0.05	6.54	-7.10	7.79E-194	602.70	-12.30
101	VAL	VAL	GLU	-0.02	4.92	13.78	1.31E-197	501.47	12.91
102	PHE	TYR	ASP	-0.05	0.72	23.39	1.40E-220	1059.29	0.23
103	ILE	VAL	GLU	-0.02	1.07	19.92	7.60E-230	443.32	17.44
104	VAL	PHE	GLU	-0.03	1.30	11.39	1.54E-230	564.98	9.41
105	ILE	PHE	GLU	-0.03	0.54	7.30	2.16E-233	556.39	6.36
106	PHE	ILE	ASP	-0.03	0.30	3.14	1.27E-258	618.16	2.81
107	VAL	MET	ASP	-0.01	0.78	-0.30	4.66E-259	616.22	-0.73
108	VAL	LEU	ASP	-0.02	2.71	14.46	8.36E-273	417.43	12.50
109	MET	TRP	GLU	-0.03	0.34	61.82	2.21E-276	823.00	39.92
