# Supplementary material for: De Novo Design and Experimental Characterization of Ultrashort Self-Associating Peptides
Source: PLoS Comput Biol. 2014 Jul 10;10(7):e1003718. doi: 10.1371/journal.pcbi.1003718 (PMC4091692; doi:10.1371/journal.pcbi.1003718)
Supplement: Table S2 — Full Stage I sequence selection results. Sequence Selection Potential Energy results were calculated for all 128 peptides possible given the design constraints and are provided here. The table is ordered by Potential Energy. (PDF) [file pcbi.1003718.s003.pdf]

Table S2: Full Stage I Sequence Selection Results. Sequence Selection Potential Energy results were calculated for all 128 peptides possible given the design constraints and are provided here. The table is ordered by Potential Energy.

Sequence	P1	P2	P3	Stage I Energy
1	TRP	TRP	ASP	-0.1255
2	TRP	TRP	GLU	-0.1193
3	TRP	TYR	GLU	-0.0912
4	TRP	TYR	ASP	-0.0851
5	TYR	TRP	GLU	-0.0801
6	TYR	TRP	ASP	-0.0788
7	TRP	LEU	GLU	-0.0755
8	TRP	LEU	ASP	-0.0687
9	LEU	TRP	ASP	-0.0664
10	LEU	TRP	GLU	-0.0663
11	TRP	PHE	ASP	-0.0625
12	LEU	LEU	GLU	-0.0618
13	TRP	PHE	GLU	-0.0605
14	TYR	PHE	GLU	-0.0565
15	TRP	MET	ASP	-0.0558
16	TRP	VAL	GLU	-0.0548
17	PHE	TRP	ASP	-0.0543
18	TRP	ALA	ASP	-0.0542
19	PHE	TYR	GLU	-0.0541
20	TRP	VAL	ASP	-0.0517
21	TYR	PHE	ASP	-0.0514
22	TRP	ILE	ASP	-0.0508
23	TYR	TYR	GLU	-0.0501
24	LEU	TYR	GLU	-0.0498
25	TRP	ALA	GLU	-0.0492
26	LEU	LEU	ASP	-0.0491
27	TYR	LEU	GLU	-0.0483
28	PHE	TYR	ASP	-0.0478
29	VAL	TRP	ASP	-0.0477
30	TRP	ILE	GLU	-0.0476
31	MET	TRP	ASP	-0.0473
32	PHE	TRP	GLU	-0.0472
33	ALA	TRP	ASP	-0.0467
34	VAL	TRP	GLU	-0.0455
35	ILE	TRP	ASP	-0.0454
36	TRP	MET	GLU	-0.0441

37	LEU	PHE	GLU	-0.0402
38	LEU	ILE	GLU	-0.0400
39	ILE	LEU	GLU	-0.0393
40	ALA	TRP	GLU	-0.0390
41	ILE	TRP	GLU	-0.0374
42	PHE	LEU	GLU	-0.0367
43	LEU	TYR	ASP	-0.0367
44	LEU	PHE	ASP	-0.0365
45	LEU	ILE	ASP	-0.0364
46	TYR	TYR	ASP	-0.0361
47	ILE	TYR	GLU	-0.0349
48	ILE	LEU	ASP	-0.0344
49	TYR	ILE	GLU	-0.0342
50	TYR	LEU	ASP	-0.0340
51	MET	PHE	ASP	-0.0332
52	VAL	TYR	GLU	-0.0331
53	PHE	MET	ASP	-0.0326
54	LEU	VAL	GLU	-0.0324
55	VAL	LEU	GLU	-0.0323
56	LEU	MET	ASP	-0.0321
57	MET	TRP	GLU	-0.0320
58	TYR	VAL	GLU	-0.0316
59	PHE	LEU	ASP	-0.0311
60	VAL	PHE	GLU	-0.0305
61	ILE	TYR	ASP	-0.0304
62	ILE	PHE	ASP	-0.0295
63	TYR	ILE	ASP	-0.0295
64	ALA	TYR	GLU	-0.0293
65	TYR	ALA	GLU	-0.0292
66	MET	LEU	ASP	-0.0292
67	VAL	PHE	ASP	-0.0291
68	LEU	ALA	GLU	-0.0284
69	PHE	ALA	ASP	-0.0281
70	ALA	PHE	ASP	-0.0281
71	ALA	LEU	GLU	-0.0278
72	MET	LEU	GLU	-0.0272
73	LEU	ALA	ASP	-0.0272
74	LEU	MET	GLU	-0.0270
75	PHE	VAL	GLU	-0.0267
76	TYR	ALA	ASP	-0.0261
77	PHE	ILE	ASP	-0.0260

78	ILE	PHE	GLU	-0.0255
79	ALA	TYR	ASP	-0.0251
80	PHE	VAL	ASP	-0.0247
81	ALA	PHE	GLU	-0.0243
82	LEU	VAL	ASP	-0.0232
83	ALA	LEU	ASP	-0.0227
84	VAL	TYR	ASP	-0.0226
85	MET	PHE	GLU	-0.0225
86	PHE	ILE	GLU	-0.0220
87	PHE	ALA	GLU	-0.0218
88	VAL	LEU	ASP	-0.0217
89	PHE	MET	GLU	-0.0211
90	ILE	ILE	ASP	-0.0211
91	TYR	VAL	ASP	-0.0208
92	VAL	VAL	GLU	-0.0199
93	VAL	ILE	GLU	-0.0173
94	ILE	VAL	GLU	-0.0172
95	VAL	ILE	ASP	-0.0170
96	ILE	ILE	GLU	-0.0166
97	MET	ILE	ASP	-0.0165
98	ILE	MET	ASP	-0.0162
99	TYR	MET	ASP	-0.0156
100	ILE	VAL	ASP	-0.0153
101	MET	TYR	ASP	-0.0151
102	PHE	PHE	ASP	-0.0150
103	ALA	ALA	ASP	-0.0150
104	VAL	ALA	ASP	-0.0133
105	VAL	VAL	ASP	-0.0126
106	VAL	MET	ASP	-0.0123
107	MET	TYR	GLU	-0.0122
108	TYR	MET	GLU	-0.0121
109	VAL	ALA	GLU	-0.0116
110	ALA	VAL	GLU	-0.0110
111	PHE	PHE	GLU	-0.0106
112	MET	ALA	ASP	-0.0103
113	MET	VAL	ASP	-0.0100
114	ALA	VAL	ASP	-0.0095
115	ALA	MET	ASP	-0.0081
116	ALA	ALA	GLU	-0.0078
117	ILE	ALA	ASP	-0.0061
118	VAL	MET	GLU	-0.0051

119	MET	VAL	GLU	-0.0048
120	MET	ILE	GLU	-0.0048
121	ALA	ILE	ASP	-0.0044
122	ILE	MET	GLU	-0.0035
123	ILE	ALA	GLU	0.0010
124	ALA	ILE	GLU	0.0016
125	MET	ALA	GLU	0.0033
126	ALA	MET	GLU	0.0050
127	MET	MET	ASP	0.0127
128	MET	MET	GLU	0.0321
