# Supplementary material for: De Novo Design and Experimental Characterization of Ultrashort Self-Associating Peptides
Source: PLoS Comput Biol. 2014 Jul 10;10(7):e1003718. doi: 10.1371/journal.pcbi.1003718 (PMC4091692; doi:10.1371/journal.pcbi.1003718)
Supplement: Table S3 — PDB structures and references for self-associating peptides. All 35 comparable PDB structures of self-associating peptides are reference. A subset of these peptides were used to compare to the crystal structure of Ac-YLD. (PDF) [file pcbi.1003718.s004.pdf]

Table S3: PDB Structures and References for Self-Associating Peptides. All 35 comparable PDB structures of self-associating peptides are reference. A subset of these peptides were used to compare to the crystal structure of Ac-YLD.

PDB ID	Chain ID	Entity ID	Sequence	Chain Length	Reference
2Y3J	A	1	1 AIIGLM	6	Colletier et al. [88]
3FOD	A	1	1 AILSST	6	Wiltzius et al. [89]
3PZZ	A	1	1 GAIIGL	6	Colletier et al. [88]
3SGS	A	1	1 GDVIEV	6	Laganowsky et al. [90]
2ONV	A	1	1 GGVVIA	6	Sawaya et al. [8]
1YJP	A	1	1 GNNQQNY	7	Nelson et al. [7]
2OMM	A	1	1 GNNQQNY	7	Sawaya et al. [8]
3NHC	A	1	1 GYMLGS	6	Apostol et al. [91]
3FPO	A	1	1 HSSNNF	6	Wiltzius et al. [89]
2Y29	A	1	1 KLVFFA	6	Colletier et al. [88]
3OW9	A	1	1 KLVFFA	6	Colletier et al. [88]
3SGO	A	1	1 KVKVLGDVIEV	11	Laganowsky et al. [90]
3LOZ	A	1	1 LSFSKD	6	Liu et al. [92]
3HYD	A	1	1 LVEALYL	7	Ivanova et al. [93]
2OMP	A	1	1 LYQLEN	6	Sawaya et al. [8]
3NVG	A	1	1 MIHFGN	6	Apostol et al. [91]
3NVE	A	1	1 MMHFGN	6	Apostol et al. [91]
2OKZ	A	1	1 MVGGVV	6	Sawaya et al. [8]
2ONA	A	1	1 MVGGVV	6	Sawaya et al. [8]
2Y3K	A	1	1 MVGGVVIA	8	Colletier et al. [88]
2Y3L	A	1	1 MVGGVVIA	8	Colletier et al. [88]
3Q2X	A	1	1 NKGAII	6	Colletier et al. [88]
3DGJ	A	1	1 NNFGAIL	7	Wiltzius et al. [94]
3FVA	A	1	1 NNQNTF	6	Wiltzius et al. [89]
2OLX	A	1	1 NNQQ	4	Sawaya et al. [8]
2ONX	A	1	1 NNQQ	4	Sawaya et al. [8]
3FTK	A	1	1 NVGSNTY	7	Wiltzius et al. [89]
3FTL	A	1	1 NVGSNTY	7	Wiltzius et al. [89]
2OL9	A	1	1 SNQNNF	6	Sawaya et al. [8]
3DG1	A	1	1 SSTNVG	6	Wiltzius et al. [94]
3FTR	A	1	1 SSTNVG	6	Wiltzius et al. [89]
2ONW	X	1	1 SSTSAA	6	Sawaya et al. [8]
2OMQ	A	1	1 VEALYL	6	Sawaya et al. [8]
2ON9	A	1	1 VQIVYK	6	Sawaya et al. [8]
3FQP	A	1	1 VQIVYK	6	Wiltzius et al. [89]
